# Supplementary figures and images for: SPARTA: Interpretable functional classification of microbiomes and detection of hidden cumulative effects
Source: PLoS Comput Biol. 2024 Nov 18;20(11):e1012577. doi: 10.1371/journal.pcbi.1012577 (PMC11611268; doi:10.1371/journal.pcbi.1012577)

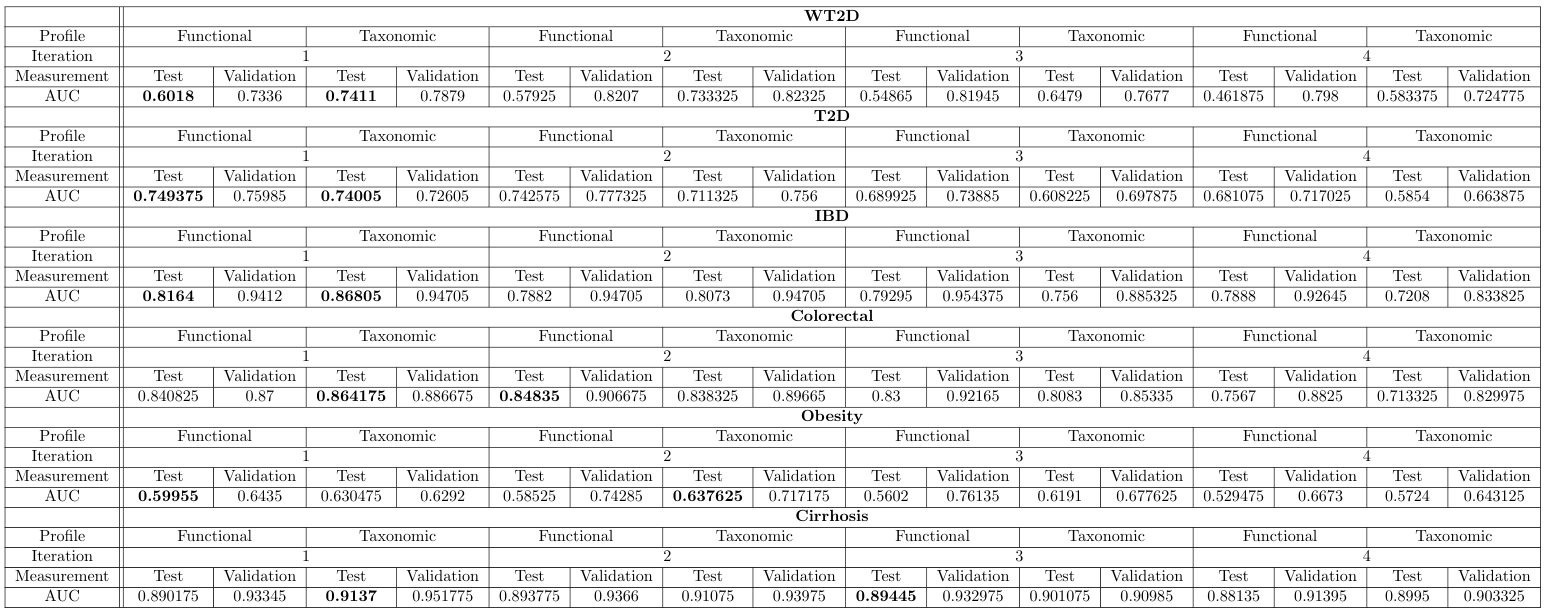

Supplement: S1 Table — The top-performing selection levels on the test sets are highlighted in bold. (PNG) [file pcbi.1012577.s006.png]

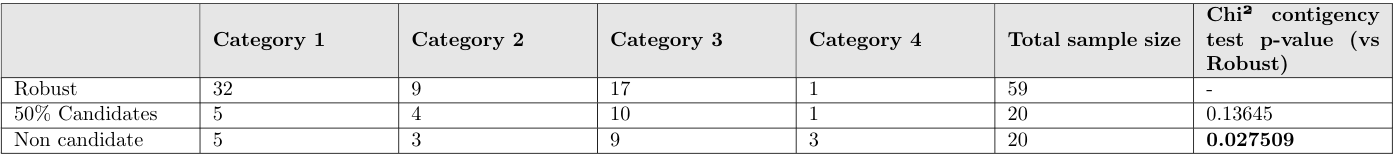

Supplement: S2 Table — (PNG) [file pcbi.1012577.s007.png]

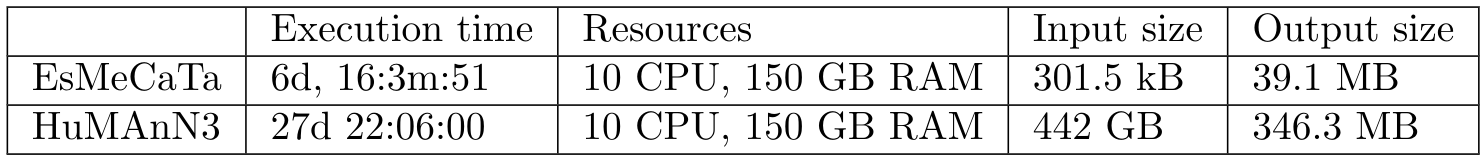

Supplement: S3 Table — (PNG) [file pcbi.1012577.s008.png]

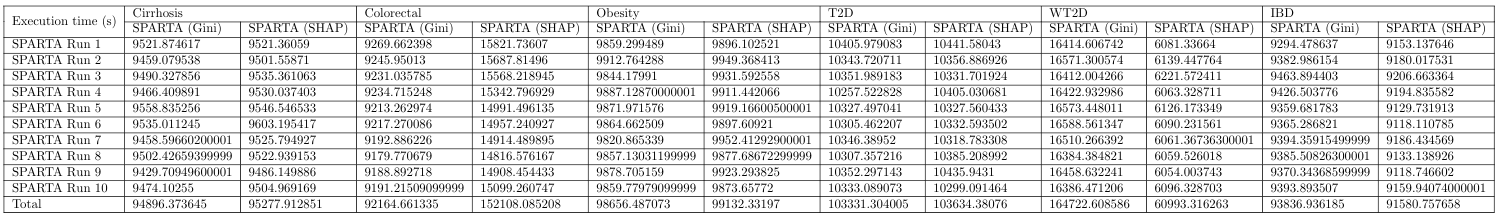

Supplement: S4 Table — (PNG) [file pcbi.1012577.s009.png]

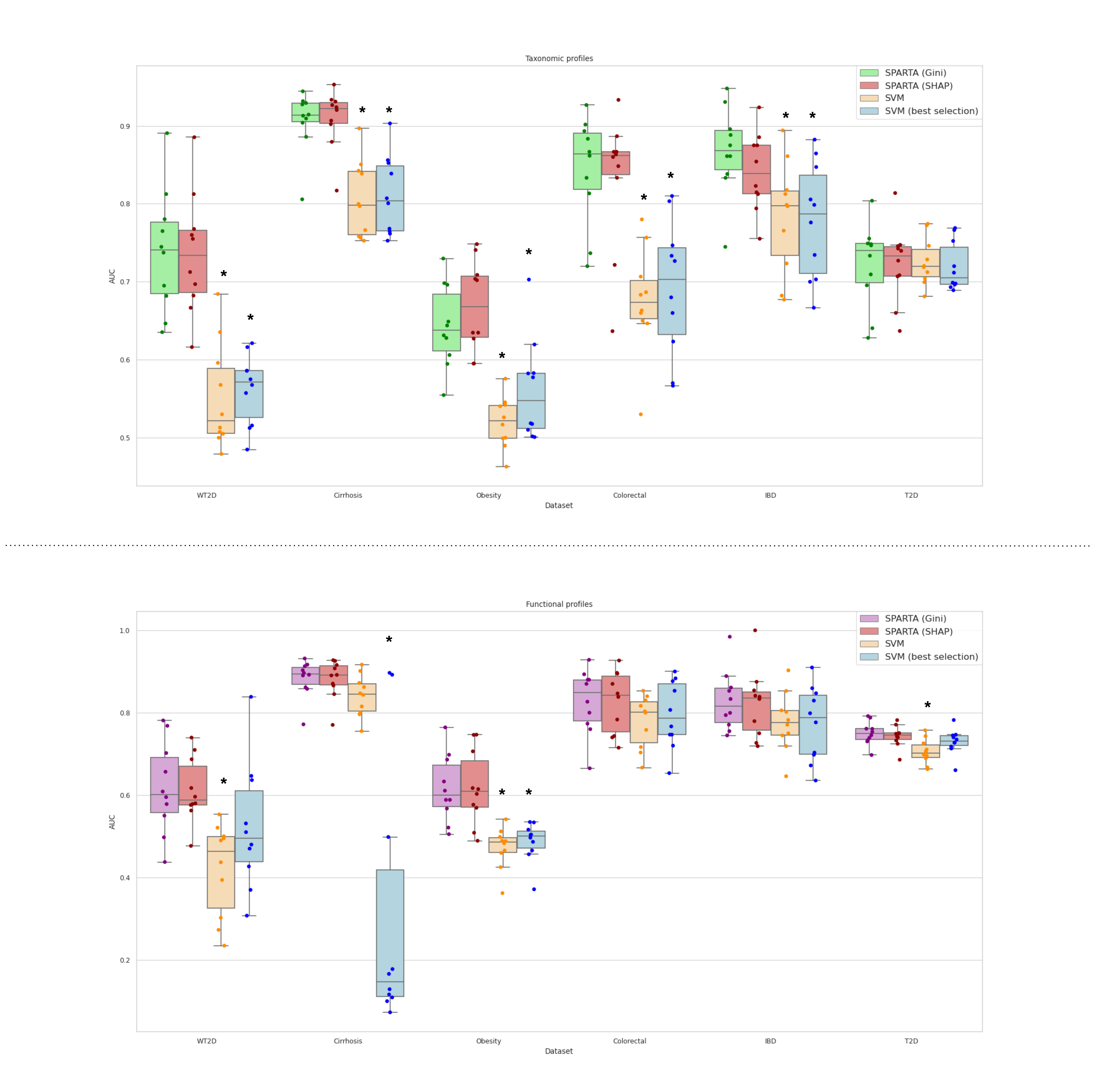

Supplement: S1 Fig — Performances at the top were obtained on the taxonomic profiles, those at the bottom were obtained on functional profiles obtained via EsMeCaTa. Similarly to Fig 2, the represented performances for the SPARTA (Gini, green for taxonomic and purple for functional, and SHAP, red) classifications are the median classification performances (AUC) for all types of profiles and each dataset, at the optimal level of selection over 10 full runs of the pipeline. SVM performances were obtained over a single run and were applied to the entire dataset (orange) or to the variable selections that correspond to the best performances for SPARTA Gini (blue). Performances obtained with SPARTA SHAP and SVMs were compared to those obtained with SPARTA Gini with a Mann-Whitney U-test. Those marked with a * showed a significant difference in distribution (p-value < 0.05). Consistent test and validation sets were used between all profiles for the classification tasks. (PNG) [file pcbi.1012577.s010.png]

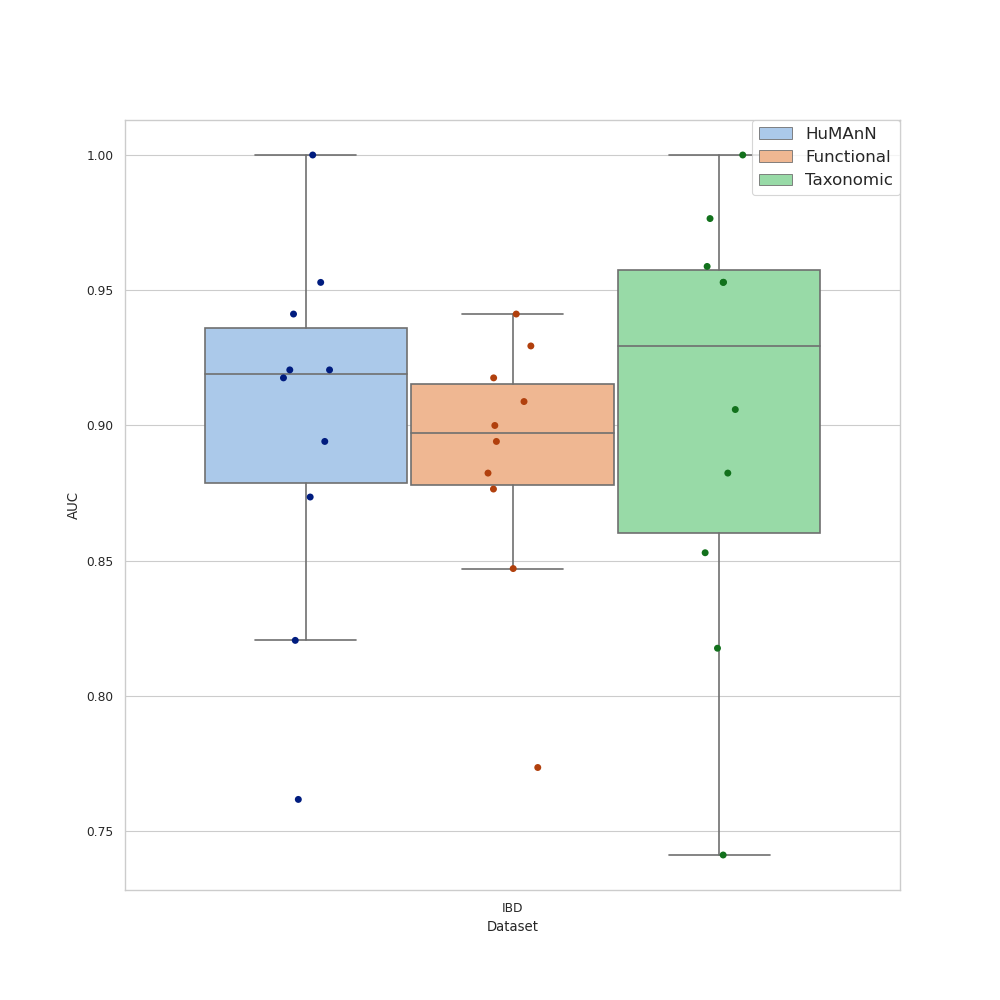

Supplement: S2 Fig — Consistent test and validation sets were used for between all profiles for the classification tasks. (PNG) [file pcbi.1012577.s011.png]

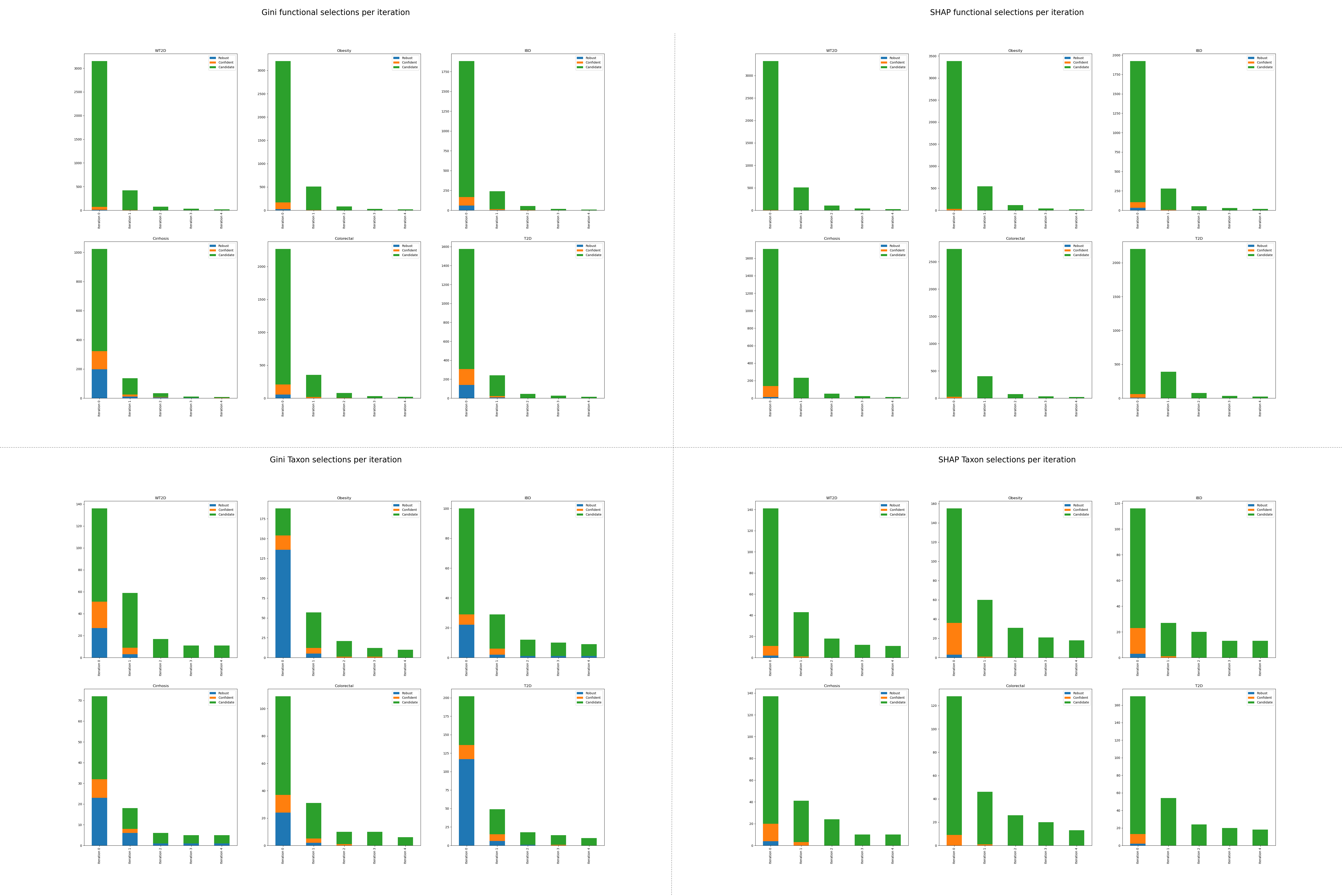

Supplement: S3 Fig — Top left: functional selections with Gini. Top right: functional selections with SHAP. Bottom left: taxonomic selections with Gini. Bottom right: taxonomic selections with SHAP. (PNG) [file pcbi.1012577.s012.png]

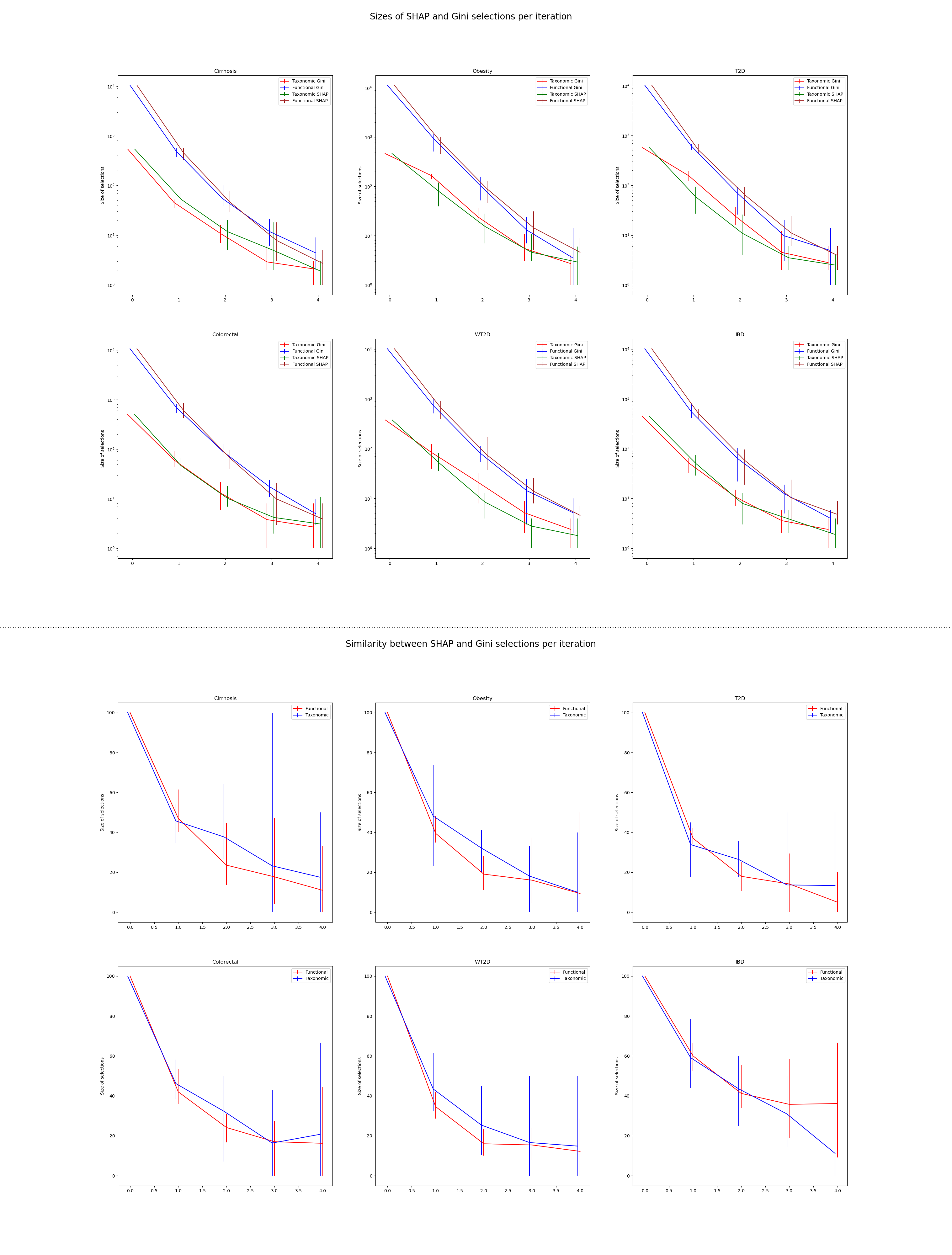

Supplement: S4 Fig — Top: sizes of the functional and taxonomic selections obtained by SPARTA with Gini and SHAP over 10 runs with 5 selective iterations, for all datasets. Bottom: similarity percentage between the individual Gini and SHAP selections, for functional and taxonomic profiles. (PNG) [file pcbi.1012577.s013.png]

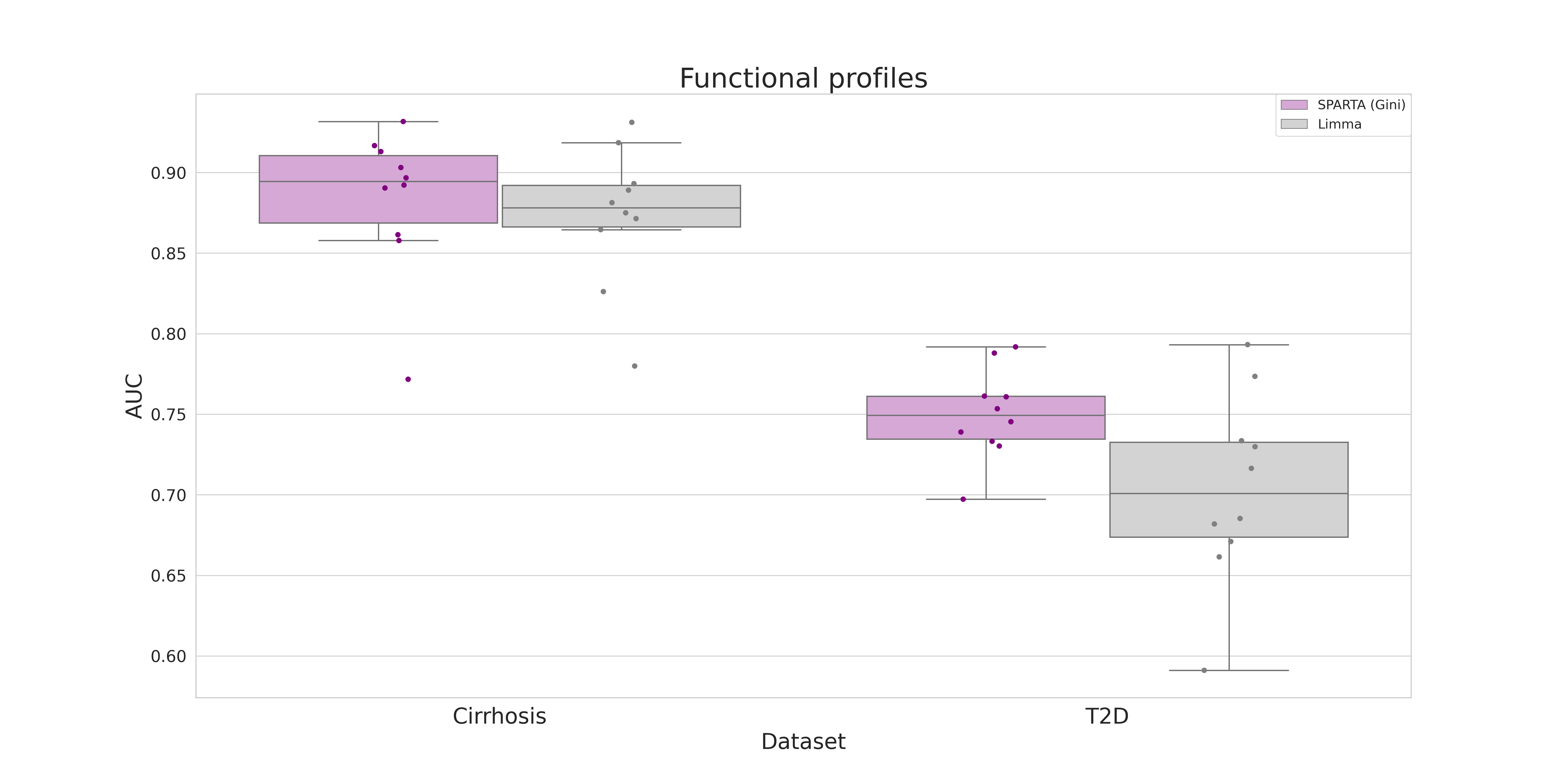

Supplement: S6 Fig — (PNG) [file pcbi.1012577.s015.png]

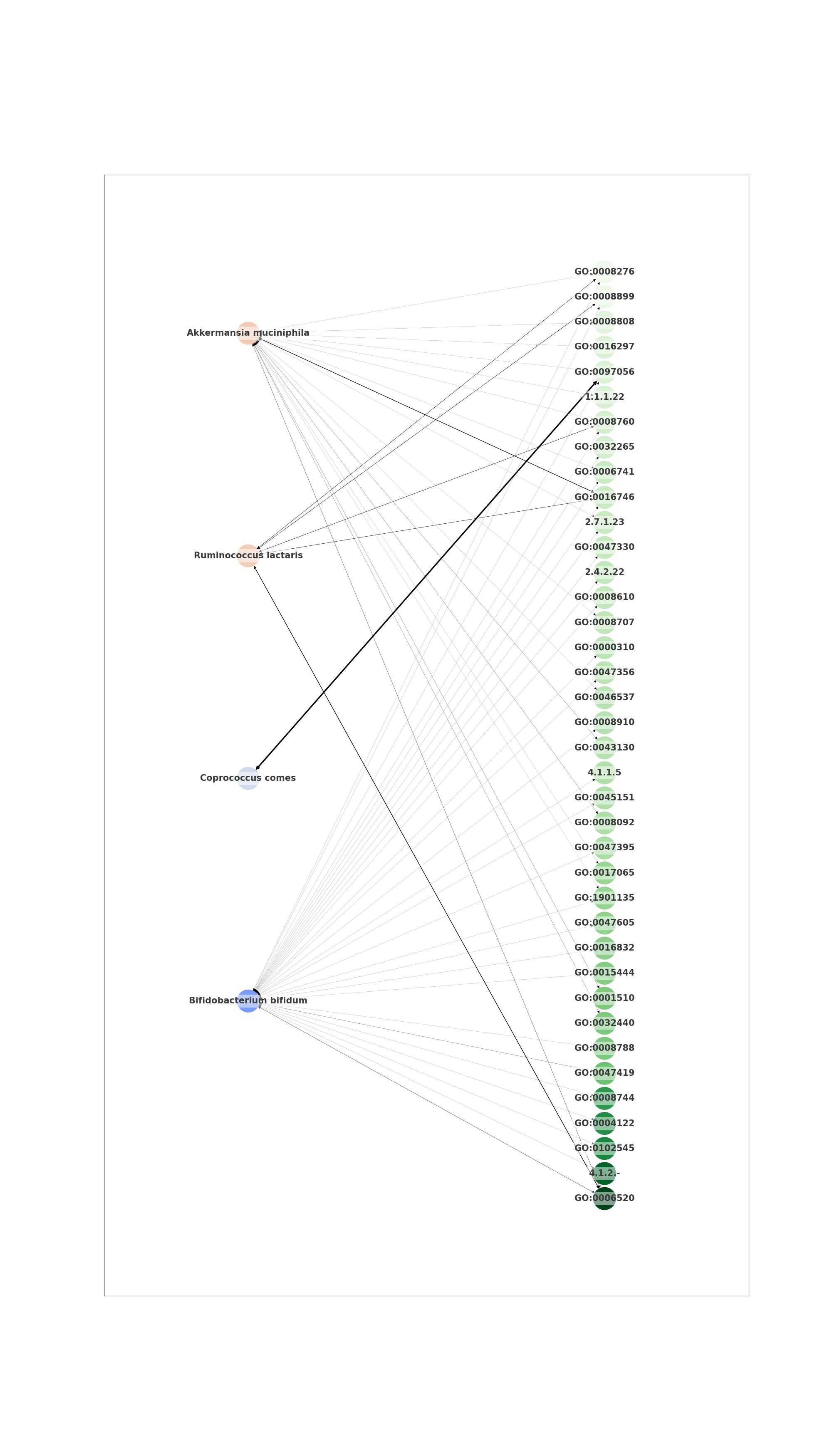

Supplement: S7 Fig — Similarly to Fig 6, the color scale for the taxa is based on their differential expression between control and unhealthy profiles, and arrow width is proportional to the strength of the taxon’s connection to the annotation. Relationships to non-robust annotations were not represented here for reasons pertaining to the readability of the figure. Represented taxa were chosen to showcase control and healthy representatives with high and low numbers of connections to robust annotations. (PNG) [file pcbi.1012577.s016.png]

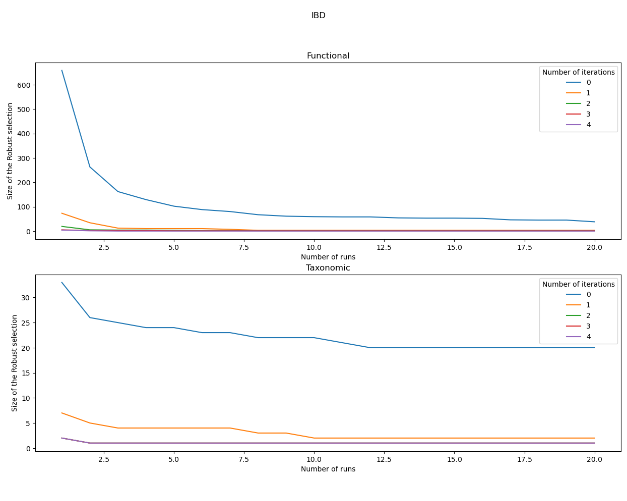

Supplement: S8 Fig — (PNG) [file pcbi.1012577.s017.png]
