# Supplementary material for: SPARTA: Interpretable functional classification of microbiomes and detection of hidden cumulative effects
Source: PLoS Comput Biol. 2024 Nov 18;20(11):e1012577. doi: 10.1371/journal.pcbi.1012577 (PMC11611268; doi:10.1371/journal.pcbi.1012577)
Supplement: Attachment — Submitted filename: Response to reviewers.pdf [file pcbi.1012577.s019.pdf]

Editor

Baptiste et al. SPARTA : Interpretable functional classification of microbiomes and detection of hidden cumulative effects The manuscript entitled" aims to provide an alternative approach to profiling enriched functions in microbiome studies using metabarcoding. The author proposes a machine learning strategy facilitated by a random forest approach. One of the main challenges in profiling functions for a given microbial community—especially without specific questions on microbial ecology or biology—is managing the trade-off between too much or too general informativeness, as highlighted by reviewer #2 in their comments on Picrust and Human. This study holds potential to offer a feasible solution to this challenge, particularly for researchers without a strong microbiology background.

We would like to start by thanking the reviewer for their comments and remarks which have helped improve the paper substantially.

Four reviewers provided constructive feedback, leading to suggestions for significant revisions. Key points include:
We have taken into account all the points mentioned below. We provide here the main contributions but all the implementations are detailed in the corresponding sections of the point-by-point review hereafter.

1. GitHub Page Improvements: Enhancements to the GitHub page to improve accessibility and usability.
We have completely reorganized the GitHub, splitting the pipeline into interoperable modules and updated the readme file accordingly. We have significantly reworked the code : SPARTA now stands as a python package, with a dedicated test suite. It can be installed with pip and all the input/output folder architecture has been refactored. We have also ensured the full reproducibility of our pipeline with parameterized seeding. Moreover, we added new ML functionalities, such as the use of SVMs in addition to RF for classification and SHAP values as a mean of importance ranking.

2. Additional Features for the Random Forest Model: incorporating more robust features into the Random Forest model.
We have implemented options for full reproducibility of the pipeline as well as options to use another measure for importance (SHAP, as suggested by reviewer 3) and added a new figure (2) to describe the algorithm which ensures the robustness of the method.

3. Comparison with DEseq2: rationals of comparisons with DEseq2 need to be better addressed.
We have implemented another option for linear variable selection, as suggested by reviewer 1, using the standard limma package. The initial results presented in the paper using DESeq2 still hold when using limma.

4. Statistical Analyses: Additional statistical analyses were also suggested.
We have implemented the option to run SVM instead of random forests (RF) as well as possibilities to pre-filter the dataset as per the user needs. As RFs outperform SVMs, we left them as the default option in SPARTA.

5. Introduction: The introduction needs substantial revision to improve clarity and coherence.
We have fully rewritten the introduction, following all the comments and suggestions for the reviewers.

6. Dataset Size and Runtime: More information regarding the size of the dataset and the time required to run SPARTA should be provided.
We have added specific benchmarks data related to these aspects in the supplementary material (Tables S3 and S4).

7. Moreover, the approach could become more useful if additional steps are included to statistically infer the dynamics of microbial communities and to profile the dynamics of microbial functions.

We added several new options to the SPARTA pipeline to give the user better flexibility regarding the statistical analysis, both before classification and post-classification.

In addition, we have also overall corrected minor typos in the main text.

Reviewer 1

Baptiste et al. present a comprehensive approach for integrating functional annotation of the gut microbiota into an automatic classification process, thereby enhancing biological interpretability. The authors proposed to use the taxonomic composition data as well as functional annotations as variables for health status classification. This work underscores the significance of understanding the functional aspects of the gut microbiota for medical applications, making the pipeline a valuable tool for microbiota feature selection. However, several questions remain to be addressed.

1. As a machine learning classifier, the SPARTA should be compared with other machine learning tools such as SVM, as mentioned in the manuscript, rather than with DEseq2.

Answer:

Thank you for this remark. Our initial choice to rely on Random Forest classifiers was based on the observation made by previous literature that they were overall the best performing classifier on microbiota data (https://doi.org/10.1371/journal.pcbi.1004977, https://doi.org/10.1038/s41598-020-63159-5, https://doi.org/10.1186/2049-2618-1-11), and their inherent capacity for variable ranking through Gini importance scores.

As suggested, we have implemented the option to run SPARTA with SVM classifiers instead of RF. This option will however only conduct classification, and not variable selection, as Gini importances are not available with this approach.

Classification results obtained from SVM classifiers on all datasets are presented in Fig S1. These models were trained on the initial datasets, as well as the SPARTA-selected subsets that gave the best performances with RF classifiers. The resulting classification performances were consistently lower than SPARTA's RF-based performances, in line with the previously cited literature. For taxonomic profiles, the gap in performance between SVM and RF models applied to the optimal SPARTA selection has been found to be significant on the basis of a Mann-Whitney U-test (alpha = 0.05) for four of the six datasets. For the functional profile, this was only the case for two of the datasets.

We edited the documentation of SPARTA to display this new option and mentioned it in the paper. We decided to keep the SVM classification results in supplementary materials for better readability of the main text.

Text changes:

- **l.32** Random Forests (RF) are particularly relevant in this regard, due to their proven efficiency in classifying microbiota data, outperforming other classic techniques such as Support Vector Machines (SVMs).
- **l.219** SPARTA, by default, trains RF [16] classifiers on the obtained functional profiles, and measures their performance in categorizing the samples. It also offers the option to train SVM classifiers.
- **l.261** SPARTA was launched with both RF and Gini importance score (default parameters), and with RF and SHAP importance score, for 10 runs, 5 selection iterations per run, and 20 trained models per iteration. We also evaluated the classification performances of SVM classifiers on the same datasets.

- **l.993** SPARTA's implementation also allows the user to classify the input data using a SVM model instead of RFs. This option will however not proceed variable selection, and can therefore only be used in single-iteration runs focused on performance.

DEseq2, intended for detecting differences in features like gene expression or taxa between groups, is not a classifier. Moreover, the authors should provide more detail on how they ran DEseq2, as the tool prefers original read count data over normalized data like relative abundance. Running Deseq2 with relative abundance may not get the best performance.

Answer:
We do agree with the reviewer that our use of DESeq2 is not standard. In order to address this issue, we have also implemented a linear selection of variables with limma [https://doi.org/10.1093/nar/gkv007]. The results are consistent with those obtained with DESeq2. With the usual threshold for the adjusted p-value (0.05), the robust annotation list from limma is empty for 4 out 6 datasets, contrary to SPARTA's automatic selection. The Cirrhosis and the T2D datasets are the only ones with non-empty robust selections. For the Cirrhosis dataset, limma provides an untractable number of robust annotations (878 FAs) while SPARTA provides 4 FAs only, which are backed by previous studies. When it comes to T2D, robust and candidate selections from limma are largely included in SPARTA's ones (only 1 annotation out of 638 is specific to limma, these others being included in SPARTA's selection, see figure 4B).
We have replaced Fig. 4 with an updated version, showing the specificities of T2D and Cirrhosis datasets and highlighting the inclusion mentioned. The other results are now in supplementary material for the sake of clarity.

Text changes :

- **l.25** Such a ranking can be handled through a linear approach, for example using the limma tool, which fits a Generalised Linear Model over the data before testing whether each variable's regression coefficient is significantly different from zero.
- **l.390** lTo explore whether SPARTA's variable selection differs from classic linear approaches, we compared our approach with a standard method designed for continuous data rather than for count data.
- **l.418** Among these datasets, Cirrhosis stands out as an outlier. Indeed, it is by far the dataset on which limma selects the most information [...] This much less restrictive threshold yields a limma selection that still largely overlaps with SPARTA's selection (74% of limma's annotations being included in SPARTA's)
- **l.507** For the Cirrhosis dataset, the SPARTA selection is the smallest of the two, however, it remains coherent with what limma selects, and yields information that is coherent with the biological question at hand.

The results do not show that the functional features improve classifier performance. In fact, the performance is reported to be "slightly inferior." This unexpected result may arise from the fact that the functional annotation is predicted from the taxonomy rather than directly derived from the sequencing data. In fact, the dataset that the authors used are all whole-genome shotgun data. The authors should consider comparing species-level taxa classifiers with gene-level functional classifiers derived from the sequence data to test if functional annotation performs better as a classifier.

Answer:
We thank the reviewer for this remark.
Using "slightly inferior", we meant that performances are not significantly different when using functional profiles and taxonomic profiles for classification, in 5 over 6 datasets. We clarified this point in the abstract.
Our choice to rely on EsMeCaTa to build functional profiles from the publicly available taxonomic profiles rather than basing ourselves on the original reads was motivated by the requirements of the latter approach

in terms of time and resources. However, as per suggested, we built a functional profile directly from the IBD dataset's raw sequences, using HuMAnN3. The process demanded 28 days of run time with 10 CPUs and 150 GB of memory, on a 442 GB input, a demand in resources that we could not meet for all datasets, explaining our restriction to the IBD example alone (see table S3 for a comparison with SPARTA). After using this as an input for SPARTA, the classification performances we obtained were very close to those obtained using the taxonomic profile, and using the functional profile derived from EsMeCaTa. These results are given in Fig S2. We added the results obtained with HuMAnN3 in the Results section, and discussed them further in the Discussion section.
In order to enhance the genericity of SPARTA's implementation, we also reworked our pipeline to be compatible with functional profiles derived from any tool of the user's preference. We have implemented the option '-fp' to directly feed SPARTA classification module with a functional profile, which can be built using any method of the user's choice. The Materials and Methods and the Git README were changed accordingly.

Text changes:

- **Abstract** This process shows that the translation of the microbiota into functional profiles gives non-significantly different performances when compared to microbial profiles on 5 of 6 datasets.
- **l.331** A comparative classification was made on the basis of a functional profile built from the raw reads of the IBD dataset with HuMAnN3, using the same parameters. The obtained results (Fig S2) show that median classification based on functional profiles built directly from the reads are on par with those obtained using EsMeCaTa, as the differences in performance are not significant based on a Mann-Whitney U-test (p-value = 0.45). Both functional profiles' performances are also non significantly different from the performance obtained on the IBD taxonomic dataset (p-value = 0.73 for HuMAnN and 0.36 for EsMeCaTa).
- **l.708** A comparison of results from our EsMeCaTa-based approach to those obtained from a profile obtained through HuMAnN3, presented in Fig S2, shows that both approaches give similar performances. However, processing patients samples with HuMAnN3 resulted in an over four-fold increase in terms of computation time, and required handling inputs of 442 GB, compared to EsMeCaTa's 302 kB entry (see Table S3). Generally, it remains an open question to choose the right trade-off between computation time, classification performance and interpretability when handling microbiota data. The modular implementation of SPARTA, allowing the user to directly specify functional profiles, aims at providing the corresponding flexibility to adjust the pipeline to the type of raw data (MGS or 16S data) or the phenotype of interest.
- **l.924** The pipeline can be launched following two steps. The first can be called with the sparta esmecata command.[...] It is possible to only give the functional table as input, in which case none of the latter three inputs would be required. The functional table given as input can be derived from sparta esmecata, or can be calculated using another tool of the user's preference. [...]
- **l.1196** A functional profile was built from the raw reads of the IBD dataset, using the HuMAnN3 tool, in context of a comparative evaluation of applications of SPARTA's classification approach to functional profiles from different sources. The process was conducted on the reads sequenced by Qin et al., available at the European Bioinformatics Institute (EBI) website with accession code ERA000116. During the process, sample V1.UC-19 could not be processed properly, resulting in a functional table devoid of this sample. As such, in Fig S2, the performances obtained on this profile were compared to classification performances obtained applying SPARTA's own functional profiling method to the IBD profile without the sample in question.

Additionally, comparing SPARTA's performance with classifiers built by the original papers could be interesting. I noticed that for the T2D dataset, the original paper achieved AUC=0.81 based on functional gene profile, higher than all SPARTA repeats. But for the cirrhosis dataset, the original paper with 15 gene markers only achieved AUC=91.8% in the training set and AUC=83.6% in the validation set, much lower than SPARTA results.

Answer:

While applied to the same datasets, the classification from DeepMicro and from SPARTA differ substantially, hence the variations in performance. First, SPARTA uses more random forests (RF) than DeepMicro initially did **(20 RF vs 5)**. Second, SPARTA explores the sources of variability to generate a more robust result, in contrast with DeepMicro. Indeed, **DeepMicro fixes a predefined seed and only describes the results with this specific seed with no exploration of the inherent variability that it could introduce.** The scope of the DeepMicro paper was focused on comparing classification performance of various approaches (RFs, SVMs, with and without projection, …) while SPARTA rather focuses on robust results.

In the context of SPARTA's first iteration on taxonomic data, we followed the same procedure as MetAML, themselves represented in DeepMicro's results, with more random forests and with several seeds. We obtained the performance results presented in Fig. 3. These performances have the same order of magnitudes as those from the original papers albeit more robust according to the design of the method. As discussed in the article, SPARTA's included variable selection is usually beneficial to classification, though this is less prevalent in application to the taxonomic datasets which are of lesser dimension to begin with.

We modified Fig. 3 and the article's text in the Results and Materials and Methods sections to clarify our position relative to the cited studies.

Text changes:

- **Abstract:** SPARTA ensures full reproducibility and exploration of inherent variability by extending state-of-the-art methods in three dimensions: increased number of trained Random Forests, selection of important variables with an iterative process, repetition of full selection process from different seeds.
- **l.78** To achieve that goal, SPARTA extends the MetAML and DeepMicro procedures in three dimensions (increased number of trained random forests, selection of important variables with an iterative process, repetition of full selection process from different seeds) to ensure full reproducibility and exploration of inherent variability in performances due to changes in training hyperparameters.
- **l.199** SPARTA is based upon the MetAML and DeepMicro procedures which describe the average results of, respectively, 20 and 5 RFs' training from a predefined seed. To gain in robustness, SPARTA trains 20 independent random forests (from a parameterized seed) to predict the patient's status. From the importance score computed on these 20 RFs, SPARTA extracts a shortlist of important features and train 20 new RFs. This procedure is then repeated on this shortlist until a drop in performance is observed (see Fig 1). This extension of MetAML procedure in 3 dimensions (20 random forests, a different seed for each of the 10 runs, an iterative process to select important variables) is a guarantee for robustness. SPARTA allows the user to set the seed for each run, ensuring full reproducibility and exploration of inherent variability in performances due to changes in training hyperparameters (see Fig 2).
- **l.273** Classification on the taxonomic datasets prior to selection is analogous to the classification without representation learning method implemented in DeepMicro, with 20 RF (SPARTA) instead of 5 (DeepMicro) and dedicated test sets (SPARTA).
- **l.301** In this case, as shown in the 'Optimal selection' column, the functional dataset gives its best performance after 2 iterations of variable selection, whereas the taxonomic dataset gives its best performance after just one. The performances of RFs trained on taxonomic and functional profiles without selection are also represented, in red and blue respectively.
- **l.1135** Therefore, SPARTA differs from DeepMicro by iterating the variable selection process: it introduces a test set - to evaluate the final performance of the model - and validation sets - to compute the performance of the RFs and derive the variable ranking for selection.

3. For all types of machine learning feature selection tools, understanding why certain features were selected is crucial. It is challenging to interpret the results, especially those from Random Forest. The authors should consider adding content on interpreting the results and possibly integrating data visualization into SPARTA to aid users in interpreting the results better.

Answer:

Indeed, interpretability of Machine Learning algorithms is a key open question. For random forests, the Gini importance index, which represents each variable's impact on optimizing the separation of samples based on their label, is the usual measure unit used for interpretability. As mentioned, its interpretation is more complex than, say, p-values associated with coefficients in linear models. Previous studies have shown the efficiency of RF applied to functional data (https://doi.org/10.1186/s40168-018-0398-3, https://doi.org/10.1093/ibd/izaa001). This suggests that this method manages to capture non-linear dependencies within the variables. Hence we chose to focus on their specific interpretability measure, the Gini index, similar to the explorations in https://doi.org/10.1186/s40168-018-0398-3, https://doi.org/10.1093/ibd/izaa001 and https://doi.org/10.1371/journal.pcbi.1004977 .

In terms of visualization of the results, we settled with an organized shortlist of the important annotations, with information pertaining to the robustness of their significance, their expression in all given profiles, and their connections to taxonomic or functional counterparts. We also tested dedicated tools such as Revigo or KEGG-mapper for visualization. However, in both cases, the resulting graphics would not provide easily interpretable results, as they were not adapted to both GO and EC nomenclatures and lacked transparency in the way the annotations were grouped together.

We do believe however that the suggested exploitation of an automatic aggregation of annotation terms for quicker exploration and better visualization is an important aspect for future research in the field.

Text changes:

- **l.869** The combination of SPARTA's outputs with a visualization method adapted for both of the employed nomenclatures, namely GO terms and EC numbers, would also be a complement to our outputs, allowing for a more intuitive exploration of their biological ramifications.

4. In the SPARTA pipeline, the authors introduce the '−i' argument and '−r' argument. It would be beneficial for the authors to test how many iterations and repeated runs are necessary to achieve a robust result, where increased repeats do not introduce new features and the feature ranking remains unchanged.

Answer:

We understand that this information would be of interest to a potential user, and that it has not been discussed in the article.

The default amount of iterations given to SPARTA, which is set to 5, has been chosen because it systematically corresponded to a drop in classification performance in our tests, and as such appeared to us as an upper limit to the amount of variable selections that should be operated on our datasets. In terms of information content, however, the obtained selection sizes per iteration and per run, plotted in Fig S4, show that the first selection is always by far the most important, and there is little variation in selection sizes past the second selection. Therefore, 2 selections could also be perceived as the upper limit by some users.

The default amount of runs, set to 10, was chosen as a compromise between robustness and computation time. Of course, a user seeking an even more stable Robust selection can opt for a higher number of runs. We checked the size of the Robust selections on the IBD dataset over 20 runs (see Fig S8) and have found that the sizes of both the functional and taxonomic Robust selections stabilize and hit a plateau after a few runs. In the case of both the functional and taxonomic profiles, 10 runs seems to be more than needed to attain a stable content for the Robust selection.

Text changes:

- **l.765** Our results highlight the need to perform at least one iteration and several repeated runs to reduce the dimensionality of the functional datasets, while maintaining the classification performance, and derive a list of robust FAs. The number of requird iterations depends on both the dataset and the user needs in terms of classification performance and interpretability. Concerning the number of iterations to perform, in this article, we presented results obtained over 10 runs, comprising 5 iterative selections each, and implemented these values as default for SPARTA. These values were chosen as a compromise between execution time and robustness of the results. Figs S4 and S8 illustrate the impact that a variation of these parameters can have on the results. When it comes to iterative selection, Fig S4 showcases that the first selection is always by far the most important, and there is little variation in selection sizes past the second selection. Therefore, 2 selections could also be perceived as the upper limit by some users, though some of our datasets have shown better classification performance beyond this level of selection. Fig S8 illustrates, in the case of the IBD dataset, that the sizes of both the functional and taxonomic Robust selections stabilize and hit a plateau after only a few runs. In both cases, 10 runs is sufficient to attain a stable content for the Robust selection. This conclusion could however only be attained *a posteriori*, once the results had been obtained. A user may want to reduce the amount of runs operated by SPARTA, but should bear in mind that these results may vary depending on the dataset. Generally speaking, SPARTA's criterion for optimal variable selection is to retain the subset that generates the best classification metric after one variable selection. An automatic test is implemented to ensure that classification performance after one iteration is not significantly lower than the one obtained with the initial dataset.

5. Is the random sampling process controlled? To achieve a reproducible analysis, it's better to have a random seed control. Or to make sure the result is robust.

Answer:
As suggested, we implemented a fully reproducible functioning of the SPARTA pipeline. By defining an initial seed, the pipeline will generate in turn a fixed sequence of random numbers, one for each run. These numbers correspond each to the "master seed" of the run. From this master seed, each run derives in a fully reproducible manner :

- A training / validation / testing split
- An initial state for the GridSearch algorithm.

We edited the git README and the article's Materials and Methods to reflect this.

Text changes:

- **l.1141** All of the described operations related to the selection of test and validation sets, and the training of RF classifiers in the context of a GridSearch algorithm, are seeded to ensure reproducibility. The initial seed can be changed to the user's discretion.

Reviewer 2

The manuscript presents an interesting workflow to classify microbiota compositions and their functional profiles using a Random Forest approach.

## Introduction
The introduction should be completely rewritten, as it fails to set the stage for the pipeline as it's presented in figure 1, and presents the biological domain of microbiome analysis with a perceived disconnection with mainstream methodologies and best practices.

Answer: We thank the reviewer for these constructive comments. We have rewritten the introduction entirely in order to integrate all of the suggestions given in this review, notably with corrections of the

confusions between vocabulary applicable to 16S and MGS and a more focused bibliography, as detailed below.

A major flaw is the ambiguity between metabarcoding (16S) and whole metagenome sequencing (WMS) approaches.

Answer *:* Both approaches are made separate and are tackled as such.

Text changes:

- **l.13** For raw shotgun metagenomic sequencing (MGS) reads, various tools have been developed for functional analysis, notably including the HuMAnN pipeline which can quantify functional annotations in a sample based on sequence alignments. For processed 16S sequencing data, PiCRUSt2 stands as one of the most popular tools for functional profiling.
- **l.75** SPARTA first retrieves the microbiota's metabolic mechanisms, regardless of the upstream sequencing method.
- **l.690** Though our manipulations were made on data derived from MGS sequencing, EsMeCaTa is also capable of processing data derived from 16S sequencing.

The literature review in the first few paragraphs feels somewhat scattered and could be tightened up to more clearly delineate previous work using taxonomic profiles versus functional profiles. The key limitations of purely taxonomic approaches should be explicitly stated upfront to better motivate the shift towards functional analyses.

Answer : The works cited in relation to this subject have been streamlined, and both the stakes expressed by the medical community, and the existing explorations of the taxonomic and functional paradigm have been summarized.

Text changes:

- **l.6** In recent years, however, some voices in the medical community have called for increased inclusion of the gut microbiota's functional paradigm in coming analyses. Specifically, taxonomy-based approaches do not properly account for functional redundancies between species and, in turn, might fall short in identifying novel biochemical pathways that should be targeted by innovative therapies.
- **l.35** While the shift to functional profiles leads to a decrease in classification performance, the subsequent analyses based on RF feature importance scores singled out impactful metabolic functions.. However, the usual number of FAs identified in biological samples (2895 ECs derived from 121 species with HuMAnN3 in context of a meta-analysis of Colorectal Cancer cohorts for example) is not easily tractable.

The general feeling is a disconnection between the introduction and the commonly used workflows and procedures, and it's not very clear how the package deals with metabarcoding or whole metagenomics sequencing. The acronyms OTU and ASV are used in metabarcoding analyses.

Text changes: This confusion has been corrected. The terms 'OTU' and 'ASV' had been used prematurely and incorrectly in the previous version of the article. The term ASV has been removed, the term OTU has been replaced in the entire text with the term "taxon"/"taxa", which is more adapted to describe the data SPARTA can take as input, as well as the data we process in this article.

The link with function is mentioned citing Picrust and Humann: it should be noted that Picrust is used with metabarcoding datasets (but it's known for the very poor performance, as it uses a taxonomic marker to

infer the function, which is basically impossible), while Humann uses whole metagenome sequencing reads, with a reasonable rationale. Mixing the two makes no sense in real life, unless the goal is to release another exercise of Machine Learning applied to biological datasets.

Answer : Both of these tools had been cited as methods to build functional profiles from sequencing data. It is true that they are not applicable to the same inputs, and that they are used in the context of entirely different sequencing approaches (16S vs MGS). Each tool's specificity has been made clear in the updated version of the introduction. Their relevance in the context of SPARTA stems from the fact the pipeline is meant to be agnostic in terms of the provenance of its input data, and could therefore be applied to data derived from 16S or MGS alike. As such, the functional profile which it calculates could be compared to either of these approaches.

Following this suggestion, SPARTA was also re-implemented to be compatible with outputs from HuMAnN3 for the classification performance assessment. We added instructions in the README file about providing HuMAnN3 outputs to SPARTA's classification module.

Text changes:

- **l.13** For raw shotgun metagenomic sequencing (MGS) reads, various tools have been developed for functional analysis, notably including the HuMAnN pipeline which can quantify functional annotations in a sample based on sequence alignments. For processed 16S sequencing data, PiCRUSt2 stands as one of the most popular tools for functional profiling.
- **l.75** SPARTA first retrieves the microbiota's metabolic mechanisms, regardless of the upstream sequencing method.
- **l.690** Though our manipulations were made on data derived from MGS sequencing, EsMeCaTa is also capable of processing data derived from 16S sequencing.
- **l.715** The modular implementation of SPARTA, allowing the user to directly specify functional profiles, aims at providing the corresponding flexibility to adjust the pipeline to the type of raw data (MGS or 16S data) or the phenotype of interest

README changes.

- If the functional profile was obtained using HuMAnN, the user should remove the partial functional abundances per associated taxon, as well as the UNMAPPED and UNINTEGRATED results.

## Package

The authors released some code in a GitHub repository (https://github.com/baptisteruiz/SPARTA). This is very far from currently accepted standards for sharing bioinformatics packages, and until these issues are fixed, this manuscript should not be accepted:

Answer: We thank the reviewer for these remarks. In order to account for these returns on SPARTA's user experience, the implementation of the pipeline has been completely reworked. As suggested, we have significantly reworked the code :

Code changes. SPARTA now stands as a python package, with a dedicated test suite. It can be installed with pip and all the input/output folder architecture has been refactored. We have also ensured the full reproducibility of our pipeline with parameterized seeding. Moreover, we added new ML functionalities, such as the use of SVMs in addition to RF for classification and SHAP values as a mean of importance ranking.

** Poor documentation: there is a general poor structure of the code and repository, to the point that there are markdown errors in the README.md. The code in `DMmodif_export_test_ver.py` is mostly commented out, generally suggesting this is a primitive development version.*

Code changes. The contents of the code were cleaned up, to remove traces of previous versions of the code which had been commented out. The README file was also refactored to include new implementations and to gain clarity.

** Lack of tests: there is no automated test suite*

Package changes. A test suite was written in complement to the new implementation, and is available on the pipeline's Git page.

** Lack of packaging: the program should be released as a Python package.*

Package changes. The pipeline was made into a pip package, to facilitate installation and to automate dependency installation.

** Lack of references: there is a directory with "Inputs", that is not documented and there is no clear reference of the source of the input files. Referring to lack of documentation, some files (such as `Label_abundance_Cirrhosis.csv`) are a bare list of values, with no practical use in real life (if someone wanted to use the package, they would need to create a chaotic set of files, making the process error prone).*

Code changes. The inputs' format and organization has been revised, notably breaking away from the rigid 'Input' folder, giving the user the choice to name the output folder, and changing the format of the Label files so that the labels are associated with the sample labels.

** Dependencies: in the paper the authors make a list of dependencies. It's trivial to add a package to the BioConda channel of Anaconda/Mamba, allowing you to install a package and its dependencies with a single command.*

Package changes. Dependency installation is now handled by pip.

## Methods
I would recommend reorganising the section.

Text changes: The Materials and Methods section has been reworked according to these suggestions.

** starting with the datasets,*

Text changes: This section has been moved to the beginning of Materials and Methods

** adding a figure describing the EsMeCaTa pipeline*

Text changes: Fig.7 has been added, and is referred to with:

- **l.924** The pipeline can be launched following two steps. The first can be called with the sparta esmecata command, represented in Fig. 7.

** Removing the parameters used by the script, using the opportunity to refactor the README where they belong*

Text changes: The pipeline's parameters are not detailed in the article anymore, and their presentation has been corrected in the Git README.

** Describing the output files (format) as they do for the input files*

Text changes:

- **l.987** The pipeline's main outputs are: the calculated functional profile, in the form of a csv table, the classification performances obtained by the pipeline through a graphical representation of the AUCs obtained at the best iteration level, and the Robust, Confident and Candidate selections obtained for each iteration level in csv files. The details of the classification performances and variable selections per run and iteration are also made available to the user.

Reviewer 2

The work describes the tool called SPARTA that focus on a machine learning application to enhance interpretable functional classification of microbiomes and the detection of hidden cumulative effects. The work is important as a way to automatize feature selection from microbiome related datasets and to give insights into connecting taxonomy information with functional annotation. Additionally, a valuable literature review was performed by the authors on the selected features. However, a significant amount of caution is needed into interpreting its results and improvements in the feature ranking approach should be made. Finally, significant improvements should be made on the Github page in order to make SPARTA actually available for the scientific community.

I really appreciate the implementation of some post-processing methods that emphasis genericity. However, ***I believe it is of utmost importance for this work to implement a more robust feature selection step before feeding the data to the Random Forest model.*** *I would like to see SPARTA to* ***allow the user to select (or not) a non-ML based method to reduce***

Answer: We thank the reviewer for this suggestion. We have added a feature to the SPARTA implementation allowing the user to enforce a filtering of variables prior to SPARTA's classification step, which allows the user to proceed to a variable selection to their discretion (i.e: filtering of low abundance variables, as suggested), and adapt it for each requested run. We would however warn that pre-filtering the input information, especially taxonomic, may not always be recommendable as our study concludes that even seemingly unimportant taxons can contribute collectively to a key metabolic function's expression in the gut microbiota.

Text Changes:

- **l.977** The user may also request that only a specified subset of the input profiles' variables be taken in account for each run. For instance, it is possible to filter out variables according to their abundance or prevalence.

Additionally, the feature ranking approach from SPARTA is one of its major selling points. Therefore, the application of only ***Gini-index is, in my opinion, insufficient. SPARTA should perform several features ranking technics (like SHAP)*** *and them suggest an approach to* ***combine them into one, more robust, rank.***

Answer: We thank the reviewer for this remark, as we had not considered SHAP importances for our initial manipulations. In the new implementations of the SPARTA pipeline, the option to use SHAP importance scores instead of Gini has been implemented, and is left to the discretion of the user. Results were obtained with a substitution of Gini by SHAP, and were added to the Supplemental Figures S1, S3 and S4.

Notably, these results show that both metrics give very similar results in terms of classification performance, with neither approach standing out as better than the other in this regard (see Fig S1). We verified that the absolute values of SHAP importances were both eligible to a variable selection using the kneebow method, as the curves of both metrics in decreasing order exhibit an inflexion point. We also confirmed that the sizes of the individual selections obtained with Gini and SHAP were consistently comparable (see Fig S4), with a few exceptions notably from the taxonomic profiles (the taxonomic Gini-based selections of Obesity and

T2D’s first selections are notably larger than their SHAP counterparts). In spite of this, the contents of the lists tended to be different: the IBD dataset’s first selections with SHAP and Gini were the most similar on average, with a mean similarity percentage of 60%. Gini-cased selections also proved to be much more robust, consistently providing larger Robust selections than SHAP-based selections, be it on the functional or taxonomic data (see Fig S3). SHAP’s Robust selections are particularly small, only exceeding 10 features in the case of IBD and Cirrhosis’ first functional selections. In both these cases, between 80 and 100% of the selected annotations, and between 50 and 100% of the selected taxons (the 50% case only accounting for 2 selected taxons) were also found in Gini’s Robust selection.

Considering all of these results, we have kept Gini importance as the default metric for SPARTA, as it proved to be more robust experimentally. The article’s contents were however adapted accordingly (see Text Changes) to account for the possibility to use SHAP in the newer versions of the pipeline and to refer to the previously discussed Supplemental results.

Text changes:

- **l.222** Finally, SPARTA generates a list of features, both taxa and FAs, which are identified as significantly discriminating between the given sample groups based on anautomatically calculated selection threshold applied to their average importance scores (Gini or SHAP values, see Materials and Methods).
- **l.261** SPARTA was launched with both RFs and Gini importance score (default parameters), and with RFs and SHAP importance score, for 10 runs, 5 selection iterations per run, and 20 trained models per iteration. We also evaluated the classification performances of SVM classifiers on the same datasets.
- **l.291** We also performed the same process evaluation with SVMs and showed that RFs consistently outperform SVMs on the datasets presented in this paper (see Fig S1), as well as RF with SHAP values as importance scores (see Fig S1, no significant difference compared to RF and Gini importance scores).
- **l.353** The sizes of the selections obtained from selections based on SHAP importances are also available in Fig S3 and S4. These results showcased that selections based on SHAP importance scores were less robust than those based on Gini importances, as the sizes of the Robust selections obtained through this method were consistently smaller than those obtained with Gini-based selection, for both functional and taxonomic profiles. Three of the functional datasets (WT2D, Obesity and Colorectal) and one functional dataset (Colorectal) even gave empty Robust selections from the first iteration with SHAP, which does not happen on any dataset with Gini. As such, only results based on Gini selections were presented here. This is however illustrative of the impact of the ranking approach on the overall quality of SPARTA's analysis.
- **l.922** SHAP (version 0.46.0)
- **l.961** This step exports a list of each trained forest’s features’ Gini or SHAP importances depending on user input
- **l.1147** These importances can be based on one of two metrics, depending on the user’s input. The first option is based on the Gini Importance metric, calculating the mean accumulation of the impurity decrease within each tree, as implemented in the Scikit-learn Python library. The other option is the SHAP importance, which calculates each variable’s contribution to a decision from the basis of a trained classifier. In our case, dealing with RFs, we relied on the SHAP package’s implementation of the TreeExplainer, which is an algorithm for calculation of SHAP values optimized for RF models. If multiple iterations of the classifier’s training are made, the feature importances are averaged over all iterations. Features are then ranked based on this metric in decreasing order. In SHAP’s case, this ranking is made based on the absolute value of the importance scores.

Major comments

1. Special attention and careful should be given to microbiome datasets.

Answer: We thank the reviewer for pointing this out. In the new version of the article, these issues are now addressed in the introduction and in the discussion, as described below.

They are usually limited in number of samples and high dimensionality.

Answer: The issue of dimensionality is indeed key in the classification of microbiota data, and is behind the rationale for variable selection in the first place. Content was added to the introduction to spotlight this topic in preparation to the description of our work.

Text changes:

- **l.38** However, the usual number of FAs identified in microbiota data (2895 ECs derived from 121 species with HuMAnN3 context of a meta-analysis of Colorectal Cancer cohorts for example) is not easily tractable, both for interpretation and for ML algorithms. As a result, the question of variable selection, that is selecting a meaningful subset among all FAs, remains a crucial post-processing step to deliver tractable results.
- **l.363** To balance the increase in information when using FAs, SPARTA operates a selection of variables on the basis of the features' importance scores. These scores, when ordered from highest to lowest, display a kink-like shape. SPARTA automatically operates a cut-off at the inflection point of the kink and probes whether classification performances are improved (see Materials and Methods). This selection aims to correct the redundancies and the dimensionality of the original dataset for better classification. It also generates one of the pipeline's main outputs: a list of ranked features (either taxa or FAs) based on their average Gini importance scores, and including an automatically computed cutoff that distinguishes significant and non-significant information. SPARTA provides the user with the list of important taxa and FAs for each iteration, the corresponding classification performance, and a focus on the best iteration after the first level of selection.

Additionally, the data generation itself has significant bias. In this sense, I recommend addressing the inherent limitations of the sequencing methodology in the abstract and further discuss it in the text, including the detection threshold of the technique and the potential for false positives/negatives, especially in cases of low abundance taxa. Additionally, it would be beneficial to succinctly describe your data preprocessing strategy in the abstract to mitigate statistical biases introduced by the method.

Answer: The biases inherent to sequencing are now included in the article's discussion. Seeing as we inherited the taxonomic profiles from previous studies, we did not apply any further preprocessing than what was already applied by the authors of MetAML and DeepMicro. The introduction and Materials and Methods were rephrased to clarify this credit.

Text changes:

- **l.84** This approach was tested on six different datasets pre-processed and used as reference for classification performance by previous works.
- **l.736** It should first be noted that there is an inherent bias to the exploitation of metagenomic data, notably concerning the taxa of lower abundance which are susceptible to be false positives.
- **l.902** The results were processed by the authors of the MetAML and DeepMicro tools as per the standard procedure described by the Human Microbiome Project, then converted to species-level relative abundance profiles via the MetaPhlAn2 tool with default parameters. Sub-species level features were then filtered using the MetAML tool.

2. Additionally, further discussion has to be regarding the annotation method. It uses the taxa information alone to then map to a reference database and infer the proteome of that community. Although this method has its benefits, it also has its cons, like database bias, proteome not necessarily targeted to the actual study environment (in this case the human gut), and the limitations of not actually being a detection of the

sample itself, which overlooks the functional variability of the species (or even strains). This should be discussed in the text.

Answer: Indeed, the cons of using EsMeCaTa for annotation were not made sufficiently clear in the article's first version. In consequence, we have added elements surrounding this question in the new version of the article's discussion, as described below. EsMeCaTa's reliance on UniProt means that any bias in the remote database would impact the tool as well, such as the inclusion of proteomes not adapted to the samples' environment of origin. The use of taxonomic profiles as an input makes the process lighter in terms of computational resources, but also make the tool reliant on the quality of the preprocessing steps, as there is no referral to the original reads.

Text changes:

- **l.698** EsMeCaTa's exploitation also comes with caveats, as its reliance on UniProt means that any bias in the remote database would impact the tool as well, such as the inclusion of proteomes not adapted to the samples' environment of origin. The use of taxonomic profiles as an input makes the process lighter in terms of computational resources, but also make the tool reliant on the quality of the preprocessing steps, as there is no referral to the original reads.

3. The authors uses only Random Forest for the classification. Although the authors tries to justify its decision, for a paper focused on a tool, it should implement other state-of-the-art approaches. Other algorithms can also extract feature importance. If the classification accuracy is in the end low, the feature importance loses part of its relevance. Therefore, aiming for a improve classification accuracy is also important.

Answer: We thank the reviewer for this remark. As mentioned in response to a similar point raised by reviewer 1, we have implemented the option to run SPARTA with three classifiers : RF&GINI, RF&SHAP and SVM. The SVM option will however only conduct classification, and not variable selection, as Gini importances are not available with this approach and SHAP values are too computing heavy.
RF & SHAP values results have been mentioned above. Classification results obtained from SVM classifiers on all datasets are presented in Fig S1. The resulting classification performances were consistently lower than SPARTA's RF-based performances, in line with the previously cited literature. For taxonomic profiles, the gap in performance between SVM and RF models applied to the optimal SPARTA selection has been found to be significant on the basis of a Mann-Whitney U-test (alpha = 0.05) for four of the six datasets. For the functional profile, this was only the case for two of the datasets.
We edited the documentation of SPARTA to display this new option and mentioned it in the paper. We decided to keep the SVM classification results in supplementary materials for better readability of the main text.

Text changes:

- **l.32** Random Forests (RF) are particularly relevant in this regard, due to their proven efficiency in classifying microbiota data, outperforming other classic techniques such as Support Vector Machines (SVMs).
- **l.219** SPARTA, by default, trains RF [16] classifiers on the obtained functional profiles, and measures their performance in categorizing the samples. It also offers the option to train SVM classifiers.
- **l.261** SPARTA was launched with both RF and Gini importance score (default parameters), and with RF and SHAP importance score, for 10 runs, 5 selection iterations per run, and 20 trained models per iteration. We also evaluated the classification performances of SVM classifiers on the same datasets.
- **l.291** We also performed the same process evaluation with SVMs and showed that RFs consistently outperform SVMs on the datasets presented in this paper (see Fig S1), as well as RF with SHAP

values as importance scores (see Fig S1, no significant difference compared to RF and Gini importance scores).
- **l.993** SPARTA's implementation also allows the user to classify the input data using a SVM model instead of RFs. This option will however not proceed variable selection, and can therefore only be used in single-iteration runs focused on performance.

4. After data acquisition, done by selecting a publicly available OTU dataset and using the functional annotation tool, the Machine learning downstream analysis of pipeline is too simple and naïve. The authors should include options for a robust preprocessing before feeding the data to the Random Forest loop.

Answer: Thank you for this suggestion. In order to allow for a preprocessing of the taxonomic and functional profiles, several aspects of SPARTA's implementation have been revised. For instance, the functional profiling step and the processing step have been decoupled, allowing the user to apply a preprocessing approach of their preference to the obtained profiles before feeding them to the latter half of the approach.
An option to enforce different variable selections for each subsequent run of the method has also been made available. This can be achieved with the command 'sparta classification --preselected_annotation preselects.csv', where preselects.csv is a csv file in which each column is named after a run and contains a list of functional annotations to be retained during said run. The same can be done with a selection of taxons. These novelties have been added to the Git README, and detailed in the article's Materials and Methods.

Text changes:
- **l.977** the user may also request that only a specified subset of the input profiles' variables be taken in account for each run. For instance, it is possible to filter out variables according to their abundance or prevalence.

5. Since the authors used previously published datasets, it is essential to include classification results from the original study, if available. Furthermore, results from cross-validation within a selected subset should be provided within the main text, particularly in cases where performance exhibits significant variability.

Answer: We thank the reviewer for this remark. As discussed in the article, SPARTA's included variable selection is usually beneficial to classification, though this is less prevalent in application to the taxonomic datasets which are of lesser dimension to begin with. As previously mentioned in answer to Reviewer 1, in the context of SPARTA's first iteration on taxonomic data, we followed the same procedure as MetAML, themselves represented in DeepMicro's results, with more random forests and with several seeds. We obtained the performance results presented in Fig. 2. These performances have the same order of magnitudes as those from the original papers albeit more robust according to the design of the method.
We modified Fig. 2 and the article's text in the Results and Materials and Methods sections to clarify our position relative to the cited studies.

We also reported the classification performance for all validation sets in table S1. Performances on the validation test are usually higher than on the test set. This is expected due to the iteration process where importance scores are averaged over all RF to perform variable selection.

Text changes:
- **l.199** SPARTA is based upon the MetAML and DeepMicro procedures which describe the average results of, respectively, 20 and 5 RFs' training from a predefined seed. In order to gain robustness, SPARTA trains 20 independent random forests (from a parameterized seed) to predict the patient's status. From the importance score computed on these 20 RFs, SPARTA extracts a short-list of important features and trains 20 new RFs. This procedure is then repeated on this shortlist until a

drop in performance is observed (see Fig 1). This extension of MetAML procedure in 3 dimensions (20 random forests, a different seed for each of the 10 runs, an iterative process to select important variables) is a guarantee for robustness. SPARTA also allows the user to set the seed for each run, ensuring full reproducibility and exploration of inherent variability in performances due to changes in training hyperparameters (see Fig 2).

- **l.294** We also reported in Table S1 the classification performances on validation sets. Those are usually higher than on the test set. This is expected due to the iteration process where importance scores are averaged over all RF to perform variable selection.
- **l.1135** Therefore, SPARTA differs from DeepMicro by iterating the variable selection process: it introduces a test set - to evaluate the final performance of the model - and validation sets - to compute the performance of the RFs and derive the variable ranking for selection.

Additionally, it is important to acknowledge instances where no features are identified as significant. This is particularly relevant given that a variable deemed important in a model with only 52% accuracy may not carry substantial meaning.

Answer: Indeed, this question would be key to a user, and should have been made clearer on our end. SPARTA's approach to variable selection is such that its selection cannot be empty: when the inflexion point of the curve of decreasing Gini importances is calculated, the feature situated at the inflexion point is kept in the Significant group, meaning that the size of the selection will always be at least of 1. In the case where there are no annotations in the Robust selection, we would advise the user to look into the Confident selection instead.
We have addressed these issues in the article's Discussion and Materials and Methods. Following this remark, a warning message has also been implemented in SPARTA to indicate to the user that the output selection may not be a good representative of the problem if the resulting AUC is 0.6 or below.

Text changes:

- **l.791** The exploration of the Confident subset or the exploitation of a lower level of selection than SPARTA's proposal could be envisioned by the user if the content included in the recommended Robust output is deemed insufficient. Users should also be mindful that the output list may not be as relevant if the classification performances are low.
- **l.984** If the best obtained median RF AUC is inferior to 0.6, a message warning that the selection may be unreliable will be passed to the user.

6. I would appreciate the inclusion of validation metrics in the "Robustness of SPARTA's feature selection: comparative evaluation" section. If feasible, please also provide a comparison of classification results using features selected by SPARTA versus those selected by DESeq.

Answer:
We have added the validation metrics in table S1 and mention them in the main text.
As suggested by reviewer 1, we have replaced DESeq with limma. In order to compare the classification performances based on SPARTA selection and limma selection, we implemented a new option in SPARTA which allows the user to specify the subset of annotations to use for classification.
Then, we have run classification on the selections obtained with Limma, presented in figure S6. The obtained results showed that Limma-based classifications were comparable to SPARTA's, though generally slightly inferior in terms of median classification, in cases when Limma's selections were not empty. However, two of the six taxonomic profiles and three of the six functional profiles included empty selections, making them at least partially incompatible with classification tasks.

Text changes:

- **l.294** We also reported in Table S1 the classification performances on validation sets. Those are usually higher than on the test set. This is expected due to the iteration process where importance scores are averaged over all RF to perform variable selection.
- **l.515** RF classification performances obtained on both selections, and presented in Fig S6, also show that limma's selections perform under SPARTA's as basis for classification, as neither of the recorded performances surpass their SPARTA counterparts.

7. *Using Abundance of functional annotation may be misleading, specifically for a reference-based annotation approach. Please discuss this further in the text.*

Answer: We thank the reviewer for bringing this up. Though the EsMeCaTa method is reference-based, the ensuing calculation of scores of functional annotations aims to convert this into a quantitative annotation-based description of the gut microbiota. As such, we felt that our scores could be viewed as an estimation of the abundance of expression of the functional annotations in a given sample. As pointed out, they are not a direct measurement of these annotations' abundance, and are dependent on the quality of the input taxonomic profile, as well as the information content of UniProt.

Text changes:

- **l.215** The prevalence of each of the obtained annotations within the individual samples and abundance data are used to calculate scores of FAs, as described in Materials and Methods. This ensures that the reference-based method EsMeCaTa provides a quantitative annotation-based description of the gut microbiota.
- **l.704** Finally, it should be noted that EsMeCaTa, being reference-based, does not provide a quantification of the functional annotations within the sample itself, as SPARTA has to rely on its own manipulation based on EsMeCaTa's results to give an estimation of the abundance of expression of these annotations.

8. *Finally, an usage resource estimation would be appreciated. It is relevant for the user to know how much memory, cpus, and time it should expect to use.*

Answer: As suggested, benchmarks of SPARTA's process have been added to the article's Methods and Supplementary Materials. We have also compared performance of HuMAnN3 and EsMeCaTa in order to estimate the trade-off between computation time, classification performance and interpretability when handling microbiota data.

Text changes:

- **l.708** A comparison of results from our EsMECaTa-based approach to those obtained from a profile obtained through HuMAnN3, presented in Fig S2, shows that both approaches give similar performances. However, processing patients samples with HuMAnN3 resulted in an over four-fold increase in terms of computation time, and required handling inputs of 442 GB, compared to EsMeCaTa's 302 kB entry (see Table S3). Generally, it remains an open question to choose the right trade-off between computation time, classification performance and interpretability when handling microbiota data. The modular implementation of SPARTA, allowing the user to directly specify functional profiles, aims at providing the corresponding flexibility to adjust the pipeline to the type of raw data (MGS or 16S data) or the phenotype of interest.
- **l.1122** The results presented in this article were obtained using 10 CPUs, and 100 GB of memory. Benchmarks of the classification process are available in Table S4 and show that SPARTA's execution time is linearly dependent on the number of requested runs.

Minor Comments

1. *Discuss more in the introduction the reasons why OTU abundances were overall more influent on disease state classification than annotations. You mention this in the introduction and it directly*

contradicts the need to then use SPARTA. Although you briefly discuss this in the discussion section, I would like to also see it in the introduction.

Answer: The main motivation regarding the use of functional annotations is to obtain a list of interpretable results, as advocated by https://doi.org/10.1016/j.tim.2017.11.002. Following studies have shown that, despite a slight drop in classification performance, functional annotation-based models have led to the discovery of key components (doi:10.1186/s40168-018-0398-3, doi:10.1093/ibd/izaa001).
We have clarified this aspect in the introduction as suggested.

Text changes:

- **l.6** In recent years, however, some voices in the medical community have called for increased inclusion of the gut microbiota's functional paradigm in coming analyses. Specifically, taxonomy-based approaches do not properly account for functional redundancies between species and, in turn, might fall short in identifying novel biochemical pathways that should be targeted by innovative therapies.
- **l.35** While the shift to functional profiles leads to a decrease in classification performance, the subsequent analyses based on RF feature importance scores singled out impactful metabolic functions.

2. The term FAs is used in the introduction but it was not defined before.

Text changes:

- **l.13** For raw shotgun metagenomic sequencing (MGS) reads, various tools have been developed for functional analysis, notably including the HuMAnN pipeline which can quantify functional annotations (FAs) in a sample based on sequence alignments.

3. Briefly explain the OTU-FA association logic in the results, as the methods are written just after.

Text changes:

- **l.609** The usage of the EsMeCaTa pipeline in building functional scores allows us to make explicit and quantify the links between taxa and their FAs, as this tool retrieves the annotations associated with a given taxon in the UniProt database.

4. The section 'Machine Learning classification performances obtained from functional profiles are similar to those from OTU profiles' are confusingly written. I would rewrite it to be clearer.

Answer : We have clarified this section, added a new paragraph outlining the method as well as produced a schematic figure (Fig 2) to support our explanations.

Text changes:

- **l.199** SPARTA is based upon the MetAML and DeepMicro procedures which describe the average results of, respectively, 20 and 5 RFs' training from a predefined seed. In order to gain robustness, SPARTA trains 20 independent random forests (from a parameterized seed) to predict the patient's status. From the importance score computed on these 20 RFs, SPARTA extracts a short-list of important features and trains 20 new RFs. This procedure is then repeated on this shortlist until a drop in performance is observed (see Fig 1). This extension of MetAML procedure in 3 dimensions (20 random forests, a different seed for each of the 10 runs, an iterative process to select important variables) is a guarantee for robustness. SPARTA also allows the user to set the seed for each run, ensuring full reproducibility and exploration of inherent variability in performances due to changes in training hyperparameters (see Fig 2).

- **l.276** For each RF trained by SPARTA, the area under the receiver operating characteristic curve (AUC) is calculated. Seeing as 20 RFs are trained within an iteration, the median of these 20 AUCs is retained to represent the performances of the iteration as a whole. The full iterative process is repeated 10 times giving 10 median performance metrics per level of iterative selection (see Fig 2). The amount of selections that leads to the highest median among these metrics is deemed to be the optimal selection, and is the one represented here for the taxonomic and functional profiles.

5. *Table 1 should include the validation metrics results*

Answer: The performances obtained on validation sets have been added to Table S1, as suggested.

6. Briefly explain in the results section definitions only explained in the material and methods. For instance, the following sentence could be improved: "To balance the afore described increase in information, SPARTA operates a selection of variables, as explained in Materials and Methods, aiming to correct issues that could stem from redundancy and dimensionality. This selection also generates one of the pipeline's main outputs: a list of ranked features (either OTUs or FAs) based on their average Gini importance scores [9], and including an automatically computed cutoff that distinguishes significant and non-significant information."

Answer: As suggested, this section has been rephrased and extended, and the Material and Methods section also extended.

Text changes:

- **l.363** To balance the increase in information when using FAs, SPARTA operates a selection of variables on the basis of the features' importance scores. These scores, when ordered from highest to lowest, display a kink-like shape. SPARTA automatically operates a cut-off at the inflection point of the kink and probes whether classification performances are improved (see Materials and Methods). This selection aims to correct the redundancies and the dimensionality of the original dataset for better classification. It also generates one of the pipeline's main outputs: a list of ranked features (either taxa or FAs) based on their average Gini importance scores, and including an automatically computed cutoff that distinguishes significant and non-significant information. SPARTA provides the user with the list of important taxa and FAs for each iteration, the corresponding classification performance, and a focus on the best iteration after the first level of selection.
- **l.784** SPARTA's criterion for optimal variable selection is to retain the subset which generates the best classification metric after one variable selection. An automatic test is implemented to ensure that classification performance after one iteration is not significantly lower than the one obtained with the initial dataset. Though this constitutes a strong basis for a first approach, previous works have also warned of it being potentially deceptive, and encouraged to investigate the significance of the evolution in performance measurements. As is, the exploration of the Confident subset or the exploitation of a lower level of selection than SPARTA's proposal could be envisioned by the user if the content included in the recommended Robust output is deemed insufficient. Similarly, a higher level of selection can be exploited if the proposed amount of Robust variables is still overwhelmingly large. Users should also be mindful that the output list may not be as relevant if the classification performances are low.

7. *Describe Fig. 4 in Figure 4. For instance, I should be able to understand what Fig 4 A and B are describing just by looking at them.*

Answer: Fig 4 has been entirely modified in the new version of the article, and its different parts are more easily differentiable now.

8. *Is the test set selected only once during the run?*

Answer: Yes, each run is characterized in part by the fact that a unique test set is put aside for it. This aspect of SPARTA's mechanisms has been rephrased in the new version's Materials and Methods.

Text changes:

- **l.949** Once per run, before any training, a subsample of the full dataset, is set aside as a test set.
- **l.972** After this, the training and variable selection step will be repeated for as many times as demanded, using the same test and validation sets as the first iteration's forests.

9. *For your SoFA equation, could you clarify what 'M' represents in the equation? It appears to denote the mechanism, but this isn't explicitly clear. As I understand, the equation involves multiplying the abundance of a given taxa (a constant number per sample) by the number of proteins associated with a specific function in a metabolic mechanism. Finally, what is the rational behind your SoFA equation.*

Answer: The equation has been clarified. Previously, *Mx* was meant to represent the taxon, but this notation became confusing due to redundancy with the notation for the *x(F,Mx)* component. The taxon has been renamed to *t* in the new formula, so as to clear this up. The rationale of the equation has also been made explicit in the text, as described below.

Text changes:

- **l.1088** As such, each annotation's SoFA is equal to the sum of the abundances of all taxa that express it, weighted by the strength of said expressions as measured by EsMeCaTa.

10. The TF-IGM is an interesting idea. You should also discuss how this approach may, or may not, influence and propagate annotation #bias in the databases.

Answer: We thank the reviewer for this remark. This potential side effect of the TF-IGM scaling was indeed not mentioned in the original version of the article. A paragraph on this issue has been added to the discussion, to warn of this potential caveat.

Text changes:

- **l.725** Finally, the scores calculated by SPARTA presented in this article are processed with the TF-IGM normalization, presented in Materials and Methods. This manipulation exacerbates the scores of the most differentially expressed annotations, heightening their highest scores, and lowering their lowest, to facilitate classification. A caveat of this approach however is that, as a cost for making the profiles more discriminating, it can enhance biases inherited from the database or from the taxonomic profiling.

11. For your cross validation, I would not do it randomly splitted, but make sure that all samples are used as training and validation at least once.

Answer: We thank the reviewer for this remark, and we fully understand that the full scope of the dataset should be used for our model training. Due to the cross-validation that is effectuated within the scope of the GridSearch which we leverage for model fine-tuning, all individuals within the training set are used for validation as well. We also checked that the randomness of the selection would be offset by the amount of repetitions of the training process, as all samples are selected to be in the validation set at least once over the total 200 RFs in practice.

Github and Using the tool:

For the tool to be used, the Github needs serious adjustments. The structure of the github should be improved. Perhaps, the authors can follow some guidelines like the Github page from the Esmecata itself.

Please include an installation procedure.

Include the version of the packages used as dependencies.

For most of the dependencies I used the instructions on the esmacata github. But it should definitely be included in the sparta github page.

Answer: As suggested by reviewer 2, we have completely reorganized the GitHub, splitting the pipeline into interoperable modules and updated the readme file accordingly. We have significantly reworked the code : SPARTA now stands as a python package, with a dedicated test suite. It can be installed with pip and all the input/output folder architecture has been refactored. We have also ensured the full reproducibility of our pipeline with parameterized seeding. Moreover, we added new ML functionalities, such as the use of SVMs in addition to RF for classification and SHAP values as a means of importance ranking.

We hope that this refactoring answers the suggestion of the reviewer since the pipeline has notably been made into a pip package, automatically handling dependencies when possible. The procedures to install any dependencies not installable through pip are described in SPARTA's new README file.

After Installing, running, failing due to missing dependencies, installing the dependencies and trying again, in a loop, I maned to run the following:

$ python main.py -d Inputs/abundance_Colorectal.txt -s relative -t tf_igm -i 3 -r 4

It returned:

Traceback (most recent call last):

File "/mnt/reviews/SPARTA/main.py", line 1649, in <module>

(...)

$ python3 -c "from ete3 import NCBITaxa; ncbi = NCBITaxa(); ncbi.update_taxonomy_database()".

I then tried again after deleting the initial intermediate files. The tool seems to be working bu requires significant computational resources. If possible, optimization should be done.

Answer: We apologize for this inconvenience. This error is due to a reference to an out-of-date NCBI database, in the context of EsMECaTa's interrogation of the UniProt database.

We implemented a '–update_ncbi' option to fix this error, which we recommend for a first use of the sparta esmecata command. This command will download the latest version of the NCBI database before proceeding to the annotation. This novelty has been added to the Git README file.

README changes:

- update_ncbi : This option allows the user to force an update of the local NCBI database (taxdump.tar.gz). This option is particularly recommended if you are running EsMeCaTa for the first time.

Reviewer 4

The manuscript "SPARTA: Interpretable Functional Classification of Microbiomes and Detection of Hidden Cumulative Effects" by Ruiz et al. introduces SPARTA (Shifting Paradigms to Annotation Representation

from Taxonomy to identify Archetypes) a computational pipeline that integrates functional annotations (FAs) into a machine learning classification and use it to classify several disease data sets. SPARTA main benefit is to keep information about the connections between taxons and annotations to be able to provide interpretability into a classification.

My main concern with the paper is that Operational Taxonomic Units (OTUs) ***consistently outperform functional annotations (FAs) in classification tasks****.*

Answer : We clarified this aspect in the text. Indeed, when performing statistical analysis, the difference in performance between OTUs and FAs used for classification appears as not statistically significant for 5 out of 6 datasets. This is consistent with previous studies (doi:10.1186/s40168-018-0398-3, doi:10.1093/ibd/izaa001).
The main advantage of switching to FA descriptors lies in the improved interpretability, as stressed in https://doi.org/10.1016/j.tim.2017.11.002, and allowed previous studies to identify relevant biological functions (doi:10.1186/s40168-018-0398-3, doi:10.1093/ibd/izaa001). We made this aspect clearer in the text, as classification performance is rather a "quality check" than an end result.

Text changes :

- **l.6** In recent years, however, some voices in the medical community have called for increased inclusion of the gut microbiota's functional paradigm in coming analyses. Specifically, taxonomy-based approaches do not properly account for functional redundancies between species and, in turn, might fall short in identifying novel biochemical pathways that should be targeted by innovative therapies.
- **l.35** While the shift to functional profiles leads to a decrease in classification performance, the subsequent analyses based on RF feature importance scores singled out impactful metabolic functions.

Even then, the paper promotes the use of functional profiles, arguing that "the innovative potential of functional profiles resides more in their prospective contribution to a biological understanding of the diseases' mechanisms than in their use for automatic classification" (line 200). However, the number of FAs required for optimal classification with SPARTA exceeds 500 (Figure 3A). The discussion subsection "Microbial and functional profiles" acknowledges ***this issue and suggests using only robust FAs.***

Answer : Indeed, we fully acknowledge the need to perform at least one iteration and several repeated runs (10 by default) in order to reduce the dimensionality of the FAs datasets, while maintaining the classification performance, and derive a list of robust functional annotations. In Fig 1, whe show that the need for such a selection process is less obvious for taxa, performance-wise. We have reimplemented SPARTA to provide the user with shortlists of the robust FAs and taxa at each iteration, as well as the corresponding classification performance. This ensures that the user can select the proper level of selection for its needs. We have clarified these aspects in the paper.

Text changes :

- **l.323** Functional profiles systematically benefit from a reduction of dimensionality, as their median performances after iterative selection (purple) are always superior to those obtained without variable selection (blue). For taxonomic profiles however, variable selection leads to a decrease in median results for three of the six datasets (Cirrhosis, WT2D,and IBD).
- **l.373** SPARTA provides the user with the list of important OTUs and FAs for each iteration, the corresponding classification performance, and a focus on the best iteration after the first level of selection.

- **l.765** Our results highlight the need to perform at least one iteration and several repeated runs in order to reduce the dimensionality of the FA datasets, while maintaining the classification performance, and derive a list of robust FAs.

  However, for the cirrhosis dataset, which shows the best classification performance, the robust subset of FAs still includes over 200 features. Given the large number of features, it seems difficult, if not impossible, to gain a clear understanding of the disease mechanisms, plus such a large number of features, makes it more challenging to provide bibliographic categorization, as done in the article for IBD.

Answer : To alleviate this issue of a potentially large number of FAs, we have modified SPARTA in order to provide, for each iteration, both the classification performances and the list of robust features. This allows the user to choose the level of selection needed, while controlling for the persistence of good performances. This has been highlighted and discussed in the main text.
As a side note, while reimplementing SPARTA to make it fully seeded and reproducible, the third iteration appeared as competitive for Cirrhosis, leading to a much more tractable set of 4 robust annotations (and 33 candidates). These 4 robust annotations are now described in the main text.

Text changes :

- **l.418** Among these datasets, Cirrhosis stands out as an outlier. Indeed, it is by far the dataset on which Limma selects the most information: on Fig 3, we can see that it selects 1550 annotations on average over 10 iterations, whereas the second highest amount, obtained with the T2D dataset, is only 26.1 on average. This also makes it the only case in which SPARTA proves to be the most stringent of the two selectors, with an average of 12 selections per run, for a Robust selection of size 4 against Limma's 878 (see Table 2). The four annotations in question are: GO:0016984 (ribulose-bisphosphate carboxylase activity), GO:0003779 (actin binding), GO:0004081 (bis(5'-nucleosyl)-tetraphosphatase (asymmetrical) activity) and GO:0018112 (proline racemase activity). Actin binding (GO:0003779) signals a participation of the gut to the maintenance of the intestinal epithellia, which plays a role in the prevention of liver diseases such as Cirrhosis. The activity of proline racemase (GO:0018112) is also indicative of proline metabolism in the gut, which has also been shown to be upregulated in cases of Cirrhosis. The activity of the bis(5'-nucleosyl)-tetraphosphatase enzyme (GO:0004081) is involved in the metabolism of both purine and pyrimidine according to KEGG, which have been found to be disturbed in mice gut during the development of Cirrhosis. Finally, ribulose-bisphosphate carboxylase (GO:0016984), though it is mostly known for its role in photosynthesis, can also be involved in the salvage of methionine, itself key in the development of liver disease.
- **l.507** For the Cirrhosis dataset, the SPARTA selection is the smallest of the two, however, it remains coherent with what limma selects, and yields information that is coherent with the biological question at hand.
- **l.373** SPARTA provides the user with the list of important OTUs and FAs for each iteration, the corresponding classification performance, and a focus on the best iteration after the first level of selection.
- **l.765** Our results highlight the need to perform at least one iteration and several repeated runs in order to reduce the dimensionality of the FA datasets, while maintaining the classification performance, and derive a list of robust FAs.

Therefore, I am not convinced that using FAs offers any advantage over OTUs.

Answer: SPARTA's main output is the selection of robust variables, which can be connected to their taxonomic or functional counterparts. As such, the more directly interpretable functional annotations appear to us to be more in line with the current demands of the medical community and hold more potential for innovation due to the cumulative effects showcased in the final parts of the article, even though they appear

to be a less efficient predictor. For these reasons, we believe them to be the most relevant result to showcase here.

A second concern is the significant variability in classification results, which suggests that SPARTA would only be useful if one already has large, well-classified datasets. In such a case, one could test if SPARTA provides good classification for a particular dataset and then use it for future classifications of the same disease. This limitation significantly narrows the applicability of the method. Moreover, as evidenced by the type 2 diabetes datasets, the results are highly dependent on the specific dataset used (WT2D vs. T2D in Figure 2).

Answer: Indeed, this is an important question. We fully agree with the reviewer. SPARTA should be retrained for each new dataset, as performances vary between cohorts, as previous studies have also shown (https://doi.org/10.1038/s41598-020-63159-5, https://doi.org/10.1371/journal.pcbi.1004977). More specifically, one of the main advantages of SPARTA lies in the identification of robust and discriminant functions within a consolidated dataset.

With regard to the broad range of classification performance between datasets, our results are consistent with previous published studies, showing that some datasets are intrinsically “easier” to handle for machine learning algorithms (https://doi.org/10.1038/s41598-020-63159-5, https://doi.org/10.1371/journal.pcbi.1004977).

When it comes to estimating the error rate of the classifiers, we ensure a “proper” training/validation/testing split to avoid any data leakage. Moreover, SPARTA’s estimation of classification performance on a given datasets relies on a retraining over 20 independent random forests (which can be further controlled with seeding, if needed). As a result, the pipeline can produce a robust estimation of the error rate for the classification task on a given dataset without the need for a second external one.

These elements were added to the article’s introduction, method and discussion, as described below.

Text changes:

- **l.37** Obtaining a set of discriminant functions is one of the major aspects when turning to functional annotations instead of taxa descriptors.
- **l.76** Then SPARTA extracts significantly discriminating features from this process, while ensuring consistent classification performances when switching from taxa to FAs as a basis for classification.
- **l.824** Classification performances in the context of FAs have been reported to be on par or slightly inferior to classification performances based on taxa. This is also consistent with our observations. As a result, current FAs-based approaches might not be best used for direct diagnostic prediction. The conditions in which a sample has been obtained, sequenced and processed most likely impacts classification performances, even for the same disease (see the differences in performance obtained in T2D and WT2D). The main advantage of current FAs-based pipelines, SPARTA included, lie in the extraction of a robust list of important FAs, related to a dataset of interest, rather than the production of a generic, directly re-usable without retraining machine-learning model.

Moreover, since the main point of the article is disease classification, there needs to be a comparison with other classification methods besides DESeq2, such as the mentioned SVMs (line 546). How well does SPARTA perform compared to other published classification methods specific to each dataset used? This comparison is crucial to evaluate the true effectiveness and relevance of SPARTA in the context of existing methodologies.

Answer: We thank the reviewer for this remark. As mentioned in response to a similar point raised by reviewers 1 and 3, we have implemented the option to run SPARTA with SVM classifiers instead of RF. This

option will however only conduct classification, and not variable selection, as Gini importances are not available with this approach.
Classification results obtained from SVM classifiers on all datasets are presented in Fig S1. The resulting classification performances were consistently lower than SPARTA's RF-based performances, in line with the previously cited literature. For taxonomic profiles, the gap in performance between SVM and RF models applied to the optimal SPARTA selection has been found to be significant on the basis of a Mann-Whitney U-test (alpha = 0.05) for four of the six datasets. For the functional profile, this was only the case for two of the datasets.
We edited the documentation of SPARTA to display this new option and mentioned it in the paper. We decided to keep the SVM classification results in supplementary materials for better readability of the main text.

Text changes:

- **l.32** RFs are also particularly relevant in this regard, due to their proven efficiency in classifying microbiota data, outperforming other classic techniques such as Support Vector Machines (SVMs).
- **l.219** SPARTA, by default, trains RF classifiers on the obtained functional profiles, and measures their performance in categorizing the samples. It also offers the option to train SVM classifiers.
- **l.261** SPARTA was launched with both RF and Gini importance score (default parameters), and with RF and SHAP importance score, for 10 runs, 5 selection iterations per run, and 20 trained models per iteration. We also evaluated the classification performances of SVM classifiers on the same datasets.
- **l.993** SPARTA's implementation also allows the user to classify the input data using a SVM model instead of RFs. This option will however not proceed variable selection, and can therefore only be used in single-iteration runs focused on performance.

One final point: my understanding is that gut microbiota can change significantly in just a matter of days due to factors such as the normal menstrual cycle (Schieren et al., Exp Clin Endocrinol Diabetes 2024) or dietary changes (David et al., Nature 2014), as well as other influences like infections and antibiotic use. Given that your datasets are based on single time points per individual, it is important to discuss how these rapid temporal changes might impact your results. Acknowledging and addressing the potential effects of such variability on the accuracy and reliability of your findings is crucial for a comprehensive understanding of the limitations and applicability of your study.

Answer: We do agree that such meta-data would help reaching a better understanding of the link between microbiota and diagnosis. As mentioned, various factors external to the disease can have an impact on the composition of the gut microbiota, and therefore induce bias in classification tasks if they are not accounted for. For this reason, we now mention that the integration of medical metadata from each individual from which a sample was taken was an important perspective for our work.
Inclusion of a fully dynamic study of the microbiota, however, appears much more complicated, as dedicated models should be trained to handle time-series. This would definitely be an interesting field to develop new methods.

Text changes:

- **l.887:** The integration of this information could also help classification, especially when they lead to a rapid and significant change in microbiota composition. For instance, the menstrual cycle, diet or antibiotic treatment could be recognized and accounted for by the models.

Here are some other minor corrections:

- *Line 66: Small typo, 'litterature'*

- *Line 169: Define AUC, it is the first time using the abbreviation. Also define it in the legend for Fig 2.*
- *Figure 3B: Include this table as a table and not a subfigure.*
- *Line 259: space missing in "adjustedp-value"*

Answer: We thank the reviewer for pointing these out. All of the listed typos and presentation defects have been corrected in the new version.

How is the IBD dataset an "average representative" of your results when it is the dataset with the second best classification performance? I would have said that T2D is more in the middle of the classifiers.

Answer: We have fully revamped the implementation of SPARTA to make it fully reproducible and easier to use. As a result, we generated again the figure presenting the classification performances, where IBD is now the third best performing dataset out of six. We have rewritten this part in the main text to clarify this aspect.
Another reason to choose the IBD was that two of the co-authors are specialized in IBD from the biological and medical standpoint. This allowed us to ensure our result were consistent with expert knowledge in the field and helped us to generate meaningful output representations.

Text changes:

- **l.521:** For the following section, we will be relying on the robust outputs from the IBD dataset as an example. These results come from the pipeline's first iteration, which are the best performing selective iterations for both profiles (see Fig 3). The IBD dataset was chosen as an illustrative representative of our results, as it is an outlier in neither classification performance, being the third best performing dataset out of six, nor in the selection of variables by limma.

Paragraph starting in line 440: I can locate the annotations in Fig 6, but I am not sure how you are going about in order in the paragraph, you say "from top to bottom" but start with the bottom (0043), then say "second and third" but point to the top and second from top to bottom. Following the order here is confusing.

Answer: We thank the reviewer for pointing this out. The figure has been remade since, and now matches the text which describes it from top to bottom.
