# Supplementary material for: SPARTA: Interpretable functional classification of microbiomes and detection of hidden cumulative effects
Source: PLoS Comput Biol. 2024 Nov 18;20(11):e1012577. doi: 10.1371/journal.pcbi.1012577 (PMC11611268; doi:10.1371/journal.pcbi.1012577)
Supplement: Attachment — Submitted filename: Answer to second reviews.pdf [file pcbi.1012577.s020.pdf]

All three reviewers considered this revision was a significant improvement from the previous submission, and brought up some minor edits for your attention. I am satisfied how you addressed our comments and suggestions. Good job!

We would like to start once again by thanking the reviewers. Their comments have greatly helped us in reworking the article. We are very happy that the results were perceived as an improvement.

Reviewer #1: The authors have addressed all my questions, and I don't have further concerns.

Reviewer #3: I would like to thank the authors for their responses to the first round of reviews. Their efforts have significantly improved the manuscript. The SPARTA resource provides a valuable entry point for machine learning analysis for microbiologists. It is also an easy-to-use solution with a more robust approach than the default parameters for feature ranking for the microbiologist audience that is not familiar with it. The manuscript addressed the comments successfully, and the GitHub was significantly improved. Currently, I have a few minor reviews to enhance the clarity of your work further:

1 - Abstract: I suggest replacing "reliable" with "consistent" when describing your solution, as it more accurately reflects the nature of your results.

Answer: The suggested vocabulary modification has been applied.

Text changes:

Abstract: "These selections were shown to be more consistent than those obtained by a state-of-the-art method"

2 - Discussion: Please include a brief discussion on why the inclusion of additional information (e.g., FA in addition to the taxonomy) might reduce model performance. More specifically, a discussion regarding the bias of the ML methods and their relationship with the dimensionality and the sampling of the dataset. This is particularly important for the biologist audience unfamiliar with the limitations of machine learning analysis.

Answer: A segment was added to the Discussion to notably clarify the dynamics between data dimensionality and model performance.

Text changes:

l.583: "Indeed, for a set amount of data, augmenting the number of variables past a certain point is known to be detrimental to model performance, and the switch to functional profiles comes with 22 times as many variables on average, without any additional samples to balance this."

3 - User guidance: To enhance reproducibility, it would be helpful to provide examples of software that can generate the input data required by SPARTA. This information could be

included in the GitHub repository. For FA profiles, it is already clear that esmecata should be used.

Answer: Thank you for this suggestion. We added information to the Git README to point users toward tools generally used to build taxonomic profiles.

Text changes:

Git README: “Such profiles can be built from MGS data, using tools such as MetaPhlan, or from 16S sequences, with QIIME2, FROGS or mothur for example. Please keep in mind that the result may require further formatting to be compatible with SPARTA.”

Additionally, in the Github, mention the Python version used. I initially tried to install Sparta in a Python 3.6 environment, but your suggested installation method (pip install -e .) only works on modern iterations of Python.

Answer: Thank you for pointing this out. We used Python 3.9 for our manipulations, therefore we have added a recommendation in the README to use SPARTA with this version.

Text changes:

Git README: “SPARTA was developed using Python 3.9. It is recommended to use this version of Python to use it as well.”

4 - The manuscript focuses on binary classification. Does SPARTA only work in binary problems? If this is indeed the case, please briefly mention this limitation in the text and provide more detailed information in the GitHub repository.

Answer: Thank you for this great remark. SPARTA should be compatible with multi-label problems, as all of its core components can handle these problems. We have made mention of this aspect of the pipeline in the Discussion.

Text changes:

l.670: “It should also be noted that in this article, only binary classification tasks were tackled. However, the key methods on which SPARTA relies are all compatible with multi-label classification tasks (SVM, RF, evaluation and importance metrics). As such, the pipeline could be compatible with such analyses.”

5 - Terminology: When referring to "Scores for FA," please ensure that you do not use "abundance" to avoid potential confusion.

Text changes: In instances where the term “abundance” was used to describe the functional profile, it was replaced with the term “score” (i.e: l.685, l.847).

6 - SVM details: When discussing the use of Support Vector Machines (SVM), please specify the hyperparameters used, particularly the kernel function as this significantly changes the SVM behavior.

Answer: The parameters of the SVM classifiers that are tuned by SPARTA are now given in Materials and Methods.

Text changes:

l.838: SVM parameters are also optimized through GridSearch, notably the regularization parameter, which tunes the impact of the loss function during training, and the classifier's kernel, which can be linear or Gaussian with Radial Basis (RBF), with a tuning of the gamma parameter (radius of each sample's area of influence) in the latter case.

7 - Future perspectives: In the discussion of potential future work, consider mentioning:
- The inclusion of other fine-tuning approaches beyond GridSearch
- The possibility of implementing new algorithms, specifically tree-based implementations tuned for the datasets (e.g., XGBoost)
-The addition of an interpretability module to describe and visualize the features

Answer: As suggested, further discussion surrounding these questions was added to the article.

Text changes:

l.698: The combination of SPARTA's outputs with a visualization method adapted for both of the employed nomenclatures, namely GO terms and EC numbers, would also be a complement to our outputs, allowing for a more intuitive exploration of their biological ramifications. A visualization such as this one could be the basis for an interpretation module for SPARTA.

l.726: "Method-wise, alternatives to the already implemented approaches could be envisioned, for example using other hyperparameter tuning methods (Bayesian Hyperparameter Optimization to replace GridSearch for example) or tree-based approaches, such as XGBoost."

8 - Terminology (L381 of the manuscripts without tracked changes): Please revise the use of the word "expressed" as this is a reference-based approach.

Text changes:

l.382: Instead of "expressed", the annotation is described as being "linked to" a total of 358 taxa.

These minor revisions will further improve the clarity and completeness of the manuscript. Once these minor reviews are addressed, I recommend the publication

Reviewer #4: The article has significantly improved since the initial draft. I particularly appreciate the new text in the "Classification methods" subsection of the Discussion. The new content provides valuable clarity. However, I recommend adding at least one sentence in the abstract to summarize the key point made in this subsection.

Answer: We modified the abstract in line with this recommendation.

Text changes:

Abstract: "This approach's main contribution however stems from its interpretability rather than its performance: through repetition, it also outputs a robust subset of discriminant variables."

One critical aspect that still requires clarification is the use of FAs for the classifier, specifically regarding the results from Figure 3. Are all candidate FAs used for this classification? If so, my original concern from the previous review remains: classification based on FAs is inferior to using taxa while the set of candidate FAs appears too large—except possibly for Cirrhosis—to provide clinicians with meaningful insights into disease characterization. Could you consider using only the Robust or at least the Confident subset of FAs for classification? If so, how does the performance compare?

Answer: Thank you for this very relevant question. Indeed, the idea of using the Robust selection for classification is a possible lead to enhance classification performance. Within SPARTA's framework however, this manipulation would be risky. Indeed, the Robust dataset is determined through 10 repetitions of the selection process, each time with a different test set put aside. This means that, through the shuffling of the test sets, almost all individuals end up influencing the training of the Random Forests at some point, and therefore variable selection. Therefore, in order to have a fair evaluation of model performance on a Robust selection without a data leak, a "super test set" would have to be set aside before running SPARTA. Otherwise, model performance will be overestimated, similarly to the case of the Validation sets described in the article.

The question of the amount of information selected by SPARTA is also important. It is difficult to define *per se* which amount of information is optimal for a comprehensive description of the issue for biologists, as there is little redundancy in our Robust lists, despite their size indeed remaining relatively large in most cases. A perspective for improvement, which is mentioned in Discussion, could be the exploitation of Semantic Web approaches to aggregate and streamline the functional information once it is obtained.

Additional minor details:

- In Figure 1, change "OTU" to "taxa," consistent with the rest of the article.

Answer: We apologize for overlooking this mistake. A new version of this figure has been made, which includes the suggested correction.

- In lines 217, 219, 743, and 867, follow "This" or "second" with a noun to clarify what is being referenced.

Text changes:

l.119: "This manipulation ensures that the reference-based method EsMeCaTa provides a quantitative annotation-based description of the gut microbiota."

l.121: "The second output consists of classification performances"

l.586: “This hypothesis is further supported by the fact that variable selection increases the functional profiles' classification performances more consistently than the taxa.”

l.706: “This observation further supports the importance of exploiting microbiota information at the functional level rather than at the taxonomic level.”

- It would be helpful to open the Discussion with a brief interpretation of the key findings.

Answer: As suggested, we have added an introductory paragraph to the Discussion section, which sums up the main elements of the Results section.

Text changes:

l.523: “Through the implementation of a new approach involving microbiota functional profiling, classification and variable selection, we have shown that the translation of the microbiota into functional profiles gives non-significantly different performances when compared to microbial profiles on 5 of 6 datasets. Through repetition, we also put forward a robust subset of discriminant variables. These selections were shown to be more reliable than those obtained by a state-of-the-art method, and their contents were validated through a manual bibliographic research on an example. The interconnections between selected taxa and functional annotations were also analyzed and revealed that important annotations emerge from the cumulated influence of non-selected taxa.”
