# Supplementary material for: SPARTA: Interpretable functional classification of microbiomes and detection of hidden cumulative effects
Source: PLoS Comput Biol. 2024 Nov 18;20(11):e1012577. doi: 10.1371/journal.pcbi.1012577 (PMC11611268; doi:10.1371/journal.pcbi.1012577)
Supplement: Attachment — Submitted filename: 02_Reviews.docx [file pcbi.1012577.s018.docx]

**Review:**

The work describes the tool called SPARTA that focus on a machine learning application to enhance interpretable functional classification of microbiomes and the detection of hidden cumulative effects. The work is important as a way to automatize feature selection from microbiome related datasets and to give insights into connecting taxonomy information with functional annotation. Additionally, a valuable literature review was performed by the authors on the selected features. However, a significant amount of caution is needed into interpreting its results and improvements in the feature ranking approach should be made. Finally, significant improvements should be made on the Github page in order to make SPARTA actually available for the scientific community.

I really appreciate the implementation of some post-processing methods that emphasis genericity. However, I believe it is of utmost importance for this work to implement a more robust feature selection step before feeding the data to the Random Forest model. I would like to see SPARTA to allow the user to select (or not) a non-ML based method to reduce dimension, like prevalence or relative abundance. Additionally, the feature ranking approach from SPARTA is one of its major selling points. Therefore, the application of only Gini-index is, in my opinion, insufficient. SPARTA should perform several features ranking technics (like SHAP) and them suggest an approach to combine them into one, more robust, rank.

**Major comments**

1. Special attention and careful should be given to microbiome datasets. They are usually limited in number of samples and high dimensionality. Additionally, the data generation itself has significant bias. In this sense, I recommend addressing the inherent limitations of the sequencing methodology in the abstract and further discuss it in the text, including the detection threshold of the technique and the potential for false positives/negatives, especially in cases of low abundance taxa. Additionally, it would be beneficial to succinctly describe your data preprocessing strategy in the abstract to mitigate statistical biases introduced by the method.
2. Additionally, further discussion has to be regarding the annotation method. It uses the taxa information alone to then map to a reference database and infer the proteome of that community. Although this method has its benefits, it also has its cons, like database bias, proteome not necessarily targeted to the actual study environment (in this case the human gut), and the limitations of not actually being a detection of the sample itself, which overlooks the functional variability of the species (or even strains). This should be discussed in the text.
3. The authors uses only Random Forest for the classification. Although the authors tries to justify its decision, for a paper focused on a tool, it should implement other state-of-the-art approaches. Other algorithms can also extract feature importance. If the classification accuracy is in the end low, the feature importance loses part of its relevance. Therefore, aiming for a improve classification accuracy is also important.
4. After data acquisition, done by selecting a publicly available OTU dataset and using the functional annotation tool, the Machine learning downstream analysis of pipeline is too simple and naïve. The authors should include options for a robust preprocessing before feeding the data to the Random Forest loop.
5. Since the authors used previously published datasets, it is essential to include classification results from the original study, if available. Furthermore, results from cross-validation within a selected subset should be provided within the main text, particularly in cases where performance exhibits significant variability. Additionally, it is important to acknowledge instances where no features are identified as significant. This is particularly relevant given that a variable deemed important in a model with only 52% accuracy may not carry substantial meaning.
6. I would appreciate the inclusion of validation metrics in the “***Robustness of SPARTA’s feature selection: comparative evaluation”*** section. If feasible, please also provide a comparison of classification results using features selected by SPARTA versus those selected by DESeq.
7. Using Abundance of functional annotation may be misleading, specifically for a reference-based annotation approach. Please discuss this further in the text.
8. Finally, an usage resource estimation would be appreciated. It is relevant for the user to know how much memory, cpus, and time it should expect to use.

**Minor Comments**

1. Discuss more in the introduction the reasons why OTU abundances were overall more influent on disease state classification than annotations. You mention this in the introduction and it directly contradicts the need to then use SPARTA. Although you briefly discuss this in the discussion section, I would like to also see it in the introduction.
2. The term FAs is used in the introduction but it was not defined before.
3. Briefly explain the OTU-FA association logic in the results, as the methods are written just after.
4. The section ‘*Machine Learning classification performances obtained from functional 165 profiles are similar to those from OTU profiles’* are confusingly written. I would rewrite it to be clearer.
5. Table 1 should include the validation metrics results
6. Briefly explain in the results section definitions only explained in the material and methods. For instance, the following sentence could be improved: *“To balance the afore described increase in information, SPARTA operates a selection of variables, as explained in Materials and Methods, aiming to correct issues that could stem from redundancy and dimensionality. This selection also generates one of the pipeline’s main outputs: a list of ranked features (either OTUs or FAs) based on their average Gini importance scores [9], and including an automatically computed cutoff that distinguishes significant and non-significant information.”*
7. Describe Fig. 4 in Figure 4. For instance, I should be able to understand what Fig 4 A and B are describing just by looking at them.
8. Is the test set selected only once during the run?
9. For your SoFA equation, could you clarify what 'M' represents in the equation? It appears to denote the mechanism, but this isn't explicitly clear. As I understand, the equation involves multiplying the abundance of a given taxa (a constant number per sample) by the number of proteins associated with a specific function in a metabolic mechanism. Finally, what is the rational behind your SoFA equation.
10. The TF-IGM is an interesting idea. You should also discuss how this approach may, or may not, influence and propagate annotation #bias in the databases.
11. For your cross validation, I would not do it randomly splitted, but make sure that all samples are used as training and validation at least once.

**Github and Using the tool:**

For the tool to be used, the Github needs serious adjustments. The structure of the github should be improved. Perhaps, the authors can follow some guidelines like the Github page from the Esmecata itself.

Please include an installation procedure.

Include the version of the packages used as dependencies.

For most of the dependencies I used the instructions on the esmacata github. But it should definitely be included in the sparta github page.

After Installing, running, failing due to missing dependencies, installing the dependencies and trying again, in a loop, I maned to run the following:

**$ python main.py -d Inputs/abundance_Colorectal.txt -s relative -t tf_igm -i 3 -r 4**

It returned:
*Traceback (most recent call last):*

*File "/mnt/reviews/SPARTA/main.py", line 1649, in <module>*

*main(args_passed)*

*File "/mnt/reviews/SPARTA/main.py", line 1449, in main*

*raise ValueError("Please give an input for "+dataset_name+".txt and Label_"+dataset_name+".csv")*

*ValueError: Please give an input for Inputs/abundance_Colorectal.txt.txt and Label_Inputs/abundance_Colorectal.txt.csv*

I then tried

**$ python main.py -d abundance_Colorectal -s relative -t tf_igm -i 3 -r 4**

Which seems to be the right command, but there is no instructions on this.

However, my results were:
*CRITICAL:esmecata.proteomes:|EsMeCaTa|proteomes| UniProt tax ID not in ete3 NCBI Taxonomy database. Try to update it with the following command: python3 -c "from ete3 import NCBITaxa; ncbi = NCBITaxa(); ncbi.update_taxonomy_database()".*

*Traceback (most recent call last):*

*File "/mnt/reviews/SPARTA/main.py", line 1649, in <module>*

*main(args_passed)*

*File "/mnt/reviews/SPARTA/main.py", line 1536, in main*

*esmecata_plus_check(esmecata_input, pipeline_path, data_ref_output_name, dataset_name, args_passed.eggnog)*

*File "/mnt/reviews/SPARTA/main.py", line 429, in esmecata_plus_check*

*count_check = proteome_check(esmecata_prot_out)*

*File "/mnt/reviews/SPARTA/main.py", line 339, in proteome_check*

*proteome_count_db = pd.read_csv(path_to_proteome_count, sep='\t')*

*File "/mnt/tools/miniconda3/envs/sparta_env/lib/python3.9/site-packages/pandas/io/parsers/readers.py", line 1026, in read_csv*

*return _read(filepath_or_buffer, kwds)*

*File "/mnt/tools/miniconda3/envs/sparta_env/lib/python3.9/site-packages/pandas/io/parsers/readers.py", line 620, in _read*

*parser = TextFileReader(filepath_or_buffer, **kwds)*

*File "/mnt/tools/miniconda3/envs/sparta_env/lib/python3.9/site-packages/pandas/io/parsers/readers.py", line 1620, in __init__*

*self._engine = self._make_engine(f, self.engine)*

*File "/mnt/tools/miniconda3/envs/sparta_env/lib/python3.9/site-packages/pandas/io/parsers/readers.py", line 1880, in _make_engine*

*self.handles = get_handle(*

*File "/mnt/tools/miniconda3/envs/sparta_env/lib/python3.9/site-packages/pandas/io/common.py", line 873, in get_handle*

*handle = open(*

*FileNotFoundError: [Errno 2] No such file or directory: '/mnt/reviews/SPARTA/EsMeCaTa_outputs/abundance_Colorectal/esmecata_outputs_proteomes/proteome_tax_id.tsv'*

I tried to run the command

**$ python3 -c "from ete3 import NCBITaxa; ncbi = NCBITaxa(); ncbi.update_taxonomy_database()".**

I then tried again after deleting the initial intermediate files. The tool seems to be working bu requires significant computational resources. If possible, optimization should be done.
